# Role of oxysterol 4β-hydroxycholesterol and liver X receptor alleles in pre-eclampsia

**DOI:** 10.1080/07853890.2025.2495763

**Published:** 2025-04-29

**Authors:** Lassi Kaartinen, Tiina Jääskeläinen, Eeva Sliz, Gamze Yazgeldi Gunaydin, Satu Wedenoja, Shintaro Katayama, Eero Kajantie, Valtteri Rinne, Seppo Heinonen, Juha Kere, Heta Merikallio, Hannele Laivuori submitted on behalf of FINNPEC group, Eeva Sliz, submitted on behalf of FinnGen group, Hannele Laivuori, Janne Hukkanen

**Affiliations:** a Research Unit of Biomedicine and Internal Medicine, University of Oulu, Oulu, Finland; b Medical Research Center Oulu, University of Oulu and Oulu University Hospital, Oulu, Finland; c Medical and Clinical Genetics, University of Helsinki and Helsinki University Hospital, University of Helsinki, Helsinki, Finland; d Department of Food and Nutrition, University of Helsinki, Helsinki, Finland; e Research Unit of Population Health, University of Oulu, Oulu, Finland; f Stem Cells and Metabolism Research Program, University of Helsinki, Helsinki, Finland; g Folkhälsan Research Center, Helsinki, Finland; h Department of Biosciences and Nutrition, Karolinska Institutet, Huddinge, Sweden; i Obstetrics and Gynecology, University of Helsinki and Helsinki University Hospital, Helsinki, Finland; j Research unit of Clinical Medicine, University of Oulu, Oulu, Finland; k Population Health Unit, Finnish Institute for Health and Welfare, Helsinki and Oulu, Oulu, Finland; l Department of Clinical and Molecular Medicine, Norwegian University of Science and Technology, Trondheim, Norway; m Admescope (Symeres Finland Ltd), Oulu, Finland; n Department of Obstetrics and Gynecology, Tampere University Hospital, The Wellbeing Services County of Pirkanmaa, Tampere, Finland; o Faculty of Medicine and Health Technology, Center for Child, Adolescent, and Maternal Health Research, Tampere University, Tampere, Finland; p Institute for Molecular Medicine Finland, Helsinki Institute of Life Science, University of Helsinki, Helsinki, Finland

**Keywords:** Liver X receptor, pre-eclampsia, 4β-hydroxycholesterol, placenta, oxysterol

## Abstract

**Background:**

Liver X receptors (LXRs) are expressed in placenta and may be associated with pre-eclampsia (PE). Oxysterols act as agonists for LXRs. We recently proposed a new blood pressure-regulating circuit with oxysterol 4β-hydroxycholesterol (4βHC) acting as a hypotensive factor *via* LXRs.

**Materials and methods:**

This study investigated the association between maternal plasma 4βHC, blood pressure (BP) indices, placental expression of LXR target genes, and patient characteristics using data from the Finnish Genetics of Pre-Eclampsia Consortium (FINNPEC) cohort. Plasma samples of 144 women with PE and 38 healthy pregnant controls as well as 44 PE and 40 control placental samples were available. In addition, genetic data from the FinnGen project was utilized to explore the associations of LXR alleles with PE and pregnancy hypertension.

**Results:**

There were no significant associations between 4βHC and BP or maternal and perinatal characteristics in FINNPEC cohort. However, plasma 4βHC was inversely correlated with the maternal body mass index. There were no associations with the genetic variants of LXRs with PE in FinnGen. LXR target genes APOD, SCARB1, TGM2, and LPCAT3 were expressed differently between PE and normal pregnancies in placental samples of FINNPEC.

**Conclusions:**

Our results demonstrate that plasma 4βHC and genetic LXR variants do not play a major role in PE and BP regulation during pregnancy. However, key LXR target genes involved in lipid metabolism were expressed differently in normal and PE pregnancies. Further research is needed to understand the complexities of oxysterols, LXRs, and their potential contributions to placental function and pregnancy outcomes.

## Key Messages

- Plasma 4βHC and genetic LXR variants do not play a major role in PE and BP regulation during pregnancy.
- Plasma 4βHC was inversely correlated with the maternal body mass index.
- The expression of LXR target genes APOD, TGM2, SCARB1 and LPCAT3 had significant difference between the PE and normal pregnancies.

## Introduction

Development of the placenta is crucial for the successful progression of pregnancy and childbirth, as an underdeveloped placenta has been associated with various pregnancy conditions, including pre-eclampsia (PE) [1]. PE is a multisystem pregnancy complication that presents itself usually with hypertension and proteinuria and can develop into a multi-organ dysfunction [2]. PE presents a significant risk for both maternal and foetal mortality. There is no treatment for PE other than delivery. Low-dose aspirin has been used to prevent or delay the onset of PE based in women who are at high risk for PE [3]. To the current knowledge, PE develops in a chain of events that first affects placental spiral arteries in early pregnancy, leading the placenta to suffer from ischemia that presumably influences endothelial response, which together enhances the formation of anti-angiogenic factors [4]. These anti-angiogenic factors induce aberrant alterations in the maternal cardiovascular system, consequently precipitating the clinical manifestations of PE. It has been found that oxidative stress through xanthine oxidoreductase may play a part in pregnancy complications such as PE [5]. Maternal immune attack toward trophoblasts that are of foreign foetal origin may play a role in initiating placental damage [6].

In early pregnancy, de novo lipogenesis is elevated as more fatty acids are needed for the basic cellular functions of the foetus [7]. Liver X receptors (LXRs) act as ligand-activated transcription factors in the nucleus and LXRs have been found to affect lipid homeostasis and atherogenesis through multiple mechanisms [8,9]. LXRs are known to stimulate de novo lipogenesis and LXR signalling elevates lipogenesis in rodents in early pregnancy [10,11]. LXRα and LXRβ are expressed in the early development of the placenta during choriovitelline and chorioallantoic placentation and can be found in the placenta throughout the pregnancy [12]. An extensive expression of LXRα is observed specifically in the amniotic membrane of the placenta [12]. LXRs have been suggested to exert a potential role in the pathophysiological progression of placental development, thereby contributing to the aetiology [13].

Oxysterols result from cholesterol oxidation, either enzymatically or by auto-oxidation [14]. Oxysterols act as agonists for LXRs [15]. However, some oxysterols have been found to directly promote inflammation in the placenta [16]. In contrast, LXR activation has reduced the inflammatory effect of oxysterols [17]. The role of the oxysterols in PE is yet unclear.

In our previous study, we proposed a novel blood pressure-regulating circuit with three components: pregnane X receptor (PXR), oxysterol 4β-hydroxycholesterol (4βHC), and LXRs [18]. PXR binds many toxins and drugs in the liver and acts as a transcription factor in the nucleus of hepatocytes [19]. We found that activation of PXR led to higher blood pressure (BP) in both rats and healthy volunteers and elevated plasma 4βHC [18,20]. However, elevated circulating 4βHC lowered systolic BP (SBP) in rats, and higher 4βHC was an independent predictor of lower SBP in humans [18]. Circulating 4βHC is a metabolite of cholesterol formed by hepatic cytochrome P450 3A4/5 (CYP3A/5), which is controlled by PXR [21]. While the precise hypotensive mechanism of 4βHC remains unknown it is known that 4βHC functions as a ligand for LXRα and LXRβ [22]. LXRs, in general, have been implicated in the regulation of BP [23].

It is well-established that during pregnancy, concentrations of 4βHC are typically higher compared to levels found in healthy non-pregnant adults [24,25]. In pregnancy with PE, serum 4βHC concentration has further been shown to be elevated compared to normal pregnancy [24]. This might be due to progesterone and many of its metabolites, known to be elevated in pregnancy, acting as agonists for PXR and inducing hepatic CYP3A [26]. Previous studies regarding pregnancy have highlighted the role of 4βHC as a measure of CYP3A activity [25]. However, the functions of 4βHC in pregnancy are yet to be explored.

The 4βHC-LXR pathway has the potential to affect placental development, possibly contributing to PE. Based on our previous discoveries regarding 4βHC and BP, and the known LXR-activating role of 4βHC, our objective was to explore the association of maternal plasma 4βHC with BP indices and maternal and perinatal characteristics. Furthermore, as studies have suggested that LXR expression in PE might differ from normal pregnancies [27,28], we also utilized the data of FinnGen to explore the association of LXR alleles with PE and pregnancy hypertension. To further explore the role of LXR pathway in PE, we conducted expression analysis of placental samples focusing on genes regulated through LXR activation. Our study provides insights to LXR-mediated effects on placental development using diverse approaches in normal and abnormal pregnancies.

## Materials and methods

### Patient data from Finnish Genetics of Pre-Eclampsia Consortium

The present study utilized data from the Finnish Genetics of Pre-Eclampsia Consortium (FINNPEC) cohort [29]. FINNPEC cohort was designed to set up a nationwide clinical and DNA database on women with and without PE, including their partners and infants, in order to identify genetic risk factors for PE. FINNPEC was collected from five Finnish university hospitals during 2008-2011 and it is a cross-sectional case-control study. The cohort consists of a total of 1450 women with PE and 1065 pregnant controls without PE. All participants provided written informed consent, including retrospective use of data. Nulliparous or multiparous women were included. PE was defined according to the American College of Obstetricians and Gynecologists 2002 criteria and the definition included hypertension (≥140 or 90 mmHg) and proteinuria (0.3 g/24 hr in urine) after 20 weeks gestation [30]. Exclusion criteria included multiple pregnancies and maternal age under 18 years. The FINNPEC Study protocol was approved by the coordinating Ethics Committee of the Hospital District of Helsinki and Uusimaa (149/EO/2007). The authors confirm that this study adheres to the principals stated in the Declaration of Helsinki.

This study used plasma samples drawn from women in the 3rd trimester (30–42 weeks) of pregnancy and stored at −80 °C until analysed. A total of 182 plasma samples from FINNPEC were available for this study. Of those, 144 plasma samples were from women with PE and 38 plasma samples were from women without PE (control group). The available samples represent a sample of convenience for the analyses. Exclusion criteria for all control women were medications for hypertension or gestational hypertension. Women with chronic hypertension prior to the pregnancy were also excluded from controls. The control group consisted of only healthy pregnant women.

### Determination of 4α-hydroxycholesterol and 4βHC in maternal plasma samples

Plasma concentrations of 4α-hydroxycholesterol (4αHC) and 4βHC were determined simultaneously by ultrahigh performance liquid chromatography coupled with high resolution mass spectrometry (UHPLC/ESI-HR-MS) at Admescope (Symeres Finland Ltd, Oulu, Finland) as described previously [31]. Plasma 4αHC levels were determined as a control for the quality of the plasma samples as 4αHC is not formed *via* CYP3A enzymes like 4βHC [32] but is the product of auto-oxidation of cholesterol. High 4αHC levels could therefore indicate degradation of the plasma due to inadequate storage conditions. In addition, plasma 4αHC as an isomer of 4βHC without LXR-activating property, was utilized as a negative control to probe the role of LXRs [32].

### Patient data from FinnGen

FinnGen is a partnership project between the public and private sectors, harnessing genetic data sourced from the Finnish biobanks [33]. This genetic data is integrated with digital health records from national hospital discharge, death, cancer and medication reimbursement registries using the national personal identification codes. In this study, data of 2223 women with ‘pre-existing hypertension complicating pregnancy, childbirth and the puerperium’ (International Classification of Diseases, 10th Revision [ICD-10] diagnoses O10.0, O10.1, O10.2, O10.3., O10.4, O10.9, and ICD-9:6420), 154 women with ‘pre-eclampsia superimposed on chronic hypertension’ (ICD-10:O11), 7212 women with ‘pre-eclampsia or eclampsia’ (ICD-10:O15) and 14 727 women with ‘pregnancy hypertension’ (ICD-10:O10, ICD-10:O11, ICD-10:O13, ICD-10:O14, ICD-10:O15, ICD-10:O16, ICD-9:642, ICD-8:63701, ICD-8:63703, ICD-8:63704, ICD-8:63709, ICD-8:63710, ICD-8:63799, ICD-8:66120) was analysed from FinnGen data freeze 9. The genetic associations were tested against up to 196,143 female controls.

Participants of FinnGen provided written informed consent for biobank research, based on the Finnish Biobank Act. Alternatively, separate research cohorts, collected prior to the Finnish Biobank Act came into effect (in September 2013) and the start of FinnGen (August 2017), were collected based on study-specific consents and later transferred to the Finnish biobanks after approval by the National Supervisory Authority for Welfare and Health. Recruitment protocols followed the biobank protocols approved by the National Supervisory Authority for Welfare and Health. The Coordinating Ethics Committee of the Hospital District of Helsinki and Uusimaa (HUS) statement number for the FinnGen study is Nr HUS/990/2017. The full ethics statement is given in the Supplement.

### Genotyping, imputation, and quality control in FinnGen

In the FinnGen project, genotyping of the samples was conducted utilizing Illumina and Affymetrix arrays (Illumina Inc., San Diego, and Thermo Fisher Scientific, Santa Clara, CA, USA). Sample quality control (QC) was performed to eliminate individuals who exhibited the following characteristics: genotype missingness (>5%), uncertain gender assignment, excessive heterozygosity (±4 SD) and non-Finnish heritage. Regarding variant QC, all variants that exhibited the following characteristics were excluded: low Hardy-Weinberg equilibrium (HWE) *p*-value (<1e-6), high missingness (>2%), and minor allele count (MAC) <3. Chip genotyped samples were pre-phased with Eagle 2.3.5 with the number of conditioning haplotypes set to 20,000. Genotype imputation was conducted using the SISu v4 reference panel, specific to the Finnish population, with Beagle 4.1 (version 08Jun17.d8b), as described in the following protocol: dx.doi.org/10.17504/protocols.io.nmndc5e. Subsequently, during post-imputation QC, variants with imputation INFO <0.6 were excluded. Genotyping, imputation and QC procedures are described in detail elsewhere [34].

### Statistical analyses

Data from FINNPEC was analysed with IBM SPSS statistics ver.29 (IBM Corp, Armonk, NY). Patient characteristics was compared with the independent samples *t*-test when numerical data was normally distributed and used the Mann–Whitney *U* test for non-normal distributions. Correlations were evaluated between maternal and perinatal characteristics, and plasma concentrations of 4αHC and 4βHC using Pearson’s test. *P*-values ≤ 0.05 were considered statistically significant.

The regenie software [35] was employed to assess the associations between genetic variants at the *NR1H3* and *NR1H2* loci (encoding LRXα and LRXβ, respectively) and the hypertensive complications of pregnancy. The association models were adjusted for factors including age, the first ten genetic principal components and the genotyping batch. Furthermore, only variants with a minimum allele count of five were included in the analysis.

### Differential gene expression analysis of placental samples

Differential gene expression analysis was performed on placental samples obtained from 44 preeclamptic and 40 normal pregnancy participants participating in the FINNPEC project. Prior to this analysis, the STRT method [36], combined with the GlobinLock method [37], was applied to these samples by Wedenoja et al. [6]. The obtained STRTseq data was preprocessed using the STRTN pipeline [38], excluding outliers identified in the quality check plots from the pipeline outputs. After preprocessing, differentially expressed genes were identified using the DESeq2 package (version 1.38.3) [39] with spike-in normalization on the R platform (version 4.2.2). The DESeq2 design formula used Condition and Library to adjust for library bias. Differential expression was defined as adjusted p-value (padj, FDR corrected) < 0.05.

In the context of LXR genes, the aliases NR1H3, NR1H2 and MYLIP were used instead of LXRα, LXRβ, and IDOL, respectively. All genes are listed in Supplementary Table 3. A total of 54 genes were initially analysed. After filtering out genes with NA-padj, the remaining 22 genes were visualized using the EnhancedVolcano package (version 1.16.0) [40]. Box plots were generated using plotCounts function (DESeq2) and ggplot2 (version 3.4.4) [41] packages to visualize the expression levels of genes with padj < 0.05, based on spike-in normalized values, with statistical significance (padj values) indicated by asterisks on the plots. Variance-stabilizing transformation was applied to normalized count data using vst function (DESeq2). The transformed expression values were then scaled and used to generate a heatmap with pheatmap package (version 1.0.12) [42].

Additionally, placental and plasma samples from the same FINNPEC cohort were used to analyse the correlation between oxysterols (4αHC and 4βHC) and LXR target gene expression. Oxysterol and gene expression data were available for 31 participants (10 normal and 21 PE pregnancies). Correlation coefficients and p-values were calculated for 22 genes and the concentrations of 4αHC and 4βHC across three groups: normal, PE and combined. Log transformation with offset were applied to the spike-in normalized gene expression levels of the 22 genes, as well as log transformation to the concentrations of 4αHC and 4βHC. Pearson’s test was used for the analysis, with *p*-values < 0.05 considered statistically significant. Fisher’s *z*-transformation was applied to assess significant differences in these correlations between normal and PE pregnancies.

## Results

### FINNPEC patient characteristics

Comparisons of the characteristics between PE and control groups of FINNPEC cohort are presented in Table 1. No difference was found in oxysterol concentrations between the control and PE groups. Plasma 4βHC had mean ratio (PE/control) of 1.15 with 95% CI of mean difference [−5.08 ng/ml, 21.38 ng/ml] between the groups and 4αHC had mean ratio (PE/control) of 1.21 with 95% CI of mean difference [−1.07 ng/ml, 8.01 ng/ml] between the groups. SBP and DBP were higher in the PE group at alltime points: BP in early pregnancy, highest BP before 20 weeks of gestation, BP at the time of sampling and maximum BP value in the gestational period (*p* < 0.001 for all). In contrast, the placental weight, child weight, the gestational age at the time of blood sampling and the gestational age at delivery were lower in the PE group than in controls (*p* < 0.001 for all).

**Table 1.** Medians (IQRs)/means ± SDs.

	PE	Controls	*P*-values
4αHC (ng/ml)	12.85 (18.82) (*n* = 144)	11.65 (15.86) (*n* = 38)	0.322
4βHC (ng/ml)	45.75 (60.40) (*n* = 144)	45.80 (45.50) (*n* = 38)	0.796
SBP early pregnancy (mmHg)	120.78 ± 10.82* (*n* = 143)	112.50 ± 8.81* (*n* = 38)	**<0.001**
DBP early pregnancy (mmHg)	77.00 (9.00) (*n* = 143)	70.50 (9.00) (*n* = 38)	**<0.001**
Highest SBP before 20 weeks of gestation (mmHg)	125.00 (16.00) (*n* = 142)	114.50 (14.00) (*n* = 38)	**<0.001**
Highest DBP before 20 weeks of gestation (mmHg)	79.00 (10.00) (*n* = 142)	71.50 (12.00) (*n* = 38)	**<0.001**
SBP at the time of sampling (mmHg)	160.00 (22.00) (*n* = 144)	120.00 (16.00) (*n* = 38)	**<0.001**
DBP at the time of sampling (mmHg)	104.00 (12.00) (*n* = 144)	76.50 (10.00) (*n* = 38)	**<0.001**
Maximum SBP (mmHg)	164.00 (18.00) (*n* = 144)	123.50 (12.00) (*n* = 38)	**<0.001**
Maximum DBP (mmHg)	109.00 (11.00) (*n* = 144)	80.00 (9.00) (*n* = 38)	**<0.001**
BP MAP 1st visit	90.82 ± 8.13* (*n* = 143)	83.64 ± 7.07* (*n* = 38)	**<0.001**
Maternal BMI at the start of the pregnancy (kg/m^2^)	23.30 (6.50) (*n* = 144)	22.90 (6.20) (*n* = 38)	0.520
Maternal BMI at term (kg/m^2^)	29.25 (6.60) (*n* = 144)	28.85 (5.00) (*n* = 38)	0.547
Birth weight of the child (g)	2893.0 (1166.0) (*n* = 144)	3723.5 (623.0) (*n* = 38)	**<0.001**
Weight of the placenta (g)	523.0 (204.0) (*n* = 138)	645.0 (245.0) (*n* = 37)	**<0.001**
Gestational age at the time of sampling (weeks)	37.0 (4.0) (*n* = 66)	39.5 (2.0) (*n* = 22)	**<0.001**
Gestational age at delivery (weeks)	37.0 (4.0) (*n* = 144)	40.0 (3.0) (*n* = 38)	**<0.001**
Maternal age at delivery (years)	30.68 ± 5.81* (*n* = 144)	31.45 ± 5.24* (*n* = 38)	0.461

Note: Data is presented as median (IQR) unless stated otherwise. Independent samples t test for normally distributed numerical data, Mann–Whitney *U* test for non-normal distribution. 4αHC 4α-hydroxycholesterol; SBP systolic blood pressure; DBP diastolic blood pressure; BMI body mass index; MAP mean arterial pressure; IQR interquartile range; SD standard deviation. *mean ± SD.

A previous study reported elevated plasma 4αHC levels in mothers, where the maximum values reached 22.2 ng/ml [43]. In this study, almost a third of samples had plasma 4αHC of over 22 ng/ml (57 of 182). Sensitivity analysis for 4βHC was performed with only the participants that had plasma 4αHC values of less than 22 ng/ml included. No significant difference between the two analyses was observed and the analyses with all the samples included are reported.

### Correlations between oxysterols and patient characteristics in FINNPEC cohort

**Figure 1.**
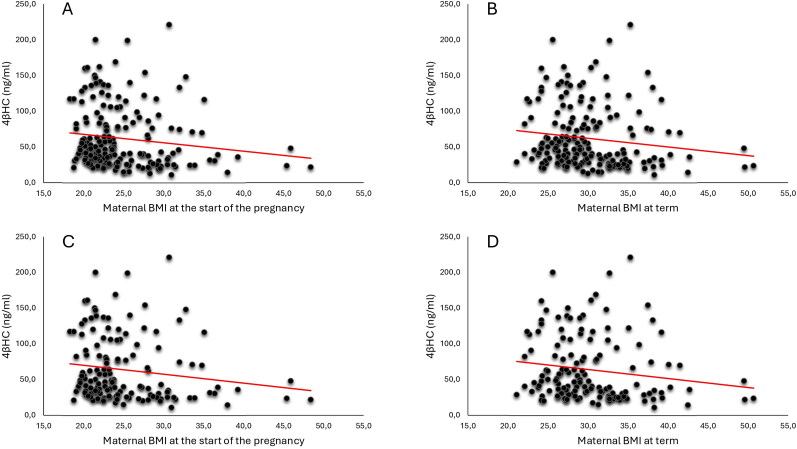
Associations of the plasma 4βHC concentration and maternal BMI. Association between plasma 4βHC concentration and maternal BMI at the start of the pregnancy with all pregnancies (A) and maternal BMI at term with all pregnancies (B). Association of the plasma 4βHC and maternal BMI at the start of the pregnancy with only PE cases (C) and with maternal BMI at term with only PE cases (D). The red trendline represents the negative correlation between the 4βHC concentrations and maternal BMI. 4βHC 4β-hydroxycholesterol; BMI body mass index 4βHC 4β-hydroxycholesterol; BMI body mass index.

In Tables 2 and 3 and Supplementary Tables 1 and 2, the correlations between plasma concentrations of 4βHC, 4αHC and patient characteristics are presented. Plasma 4βHC correlated negatively with the maternal BMI at the start of the pregnancy and at term (*p* = 0.008 and *p* = 0.003, respectively, Table 3) in all pregnancies. The negative correlation between plasma 4βHC and BMI at the start of the pregnancy and at term was more pronounced in the PE group (*p* = 0.014 and *p* = 0.006, respectively) but not statistically significant when analysed in the control group only. Correlations between 4βHC and BMI are presented in Figure 1. Plasma 4βHC correlated positively with maternal age at term in all pregnancies (*p* = 0.020), but this was not detected when the control or PE groups were analysed separately. Plasma 4βHC and 4αHC correlated negatively with the gestational age at the time of blood sampling in all pregnancies (*p* = 0.015 and *p* = 0.008, respectively). There were no significant associations between plasma 4βHC or 4αHC and maternal weight gain, weight of placenta, and birth weight of the child.

**Table 2.** Correlations of 4βHC and blood pressure.

	SBP early pregnancy	DBP early pregnancy	Highest SBP before 20 weeks of gestation	Highest DBP before 20 weeks of gestation	SBP at the time of sampling	DBP at the time of sampling	BP MAP 1st visit
Normal pregnancies 4βHC	0.171 (*p* = 0.31) *n* = 38	−0.107 (*p* = 0.52) *n* = 38	−0.052 (*p* = 0.76) *n* = 38	−0.117 (*p* = 0.49) *n* = 38	−0.016 (*p* = 0.93) *n* = 38	−0.014 (*p* = 0.93) *n* = 38	−0.053 (*p* = 0.75) *n* = 38
Pre-eclampsia 4βHC	0.026 (*p* = 0.76) *n* = 143	0.018 (*p* = 0.83) *n* = 143	0.004 (*p* = 0.96) *n* = 142	0.062 (*p* = 0.46) *n* = 142	−0.049 (*p* = 0.56) *n* = 144	−0.023 (*p* = 0.78) *n* = 144	0.025 (*p* = 0.77) *n* = 143
All pregnancies 4βHC	0.052 (*p* = 0.49) *n* = 181	0.006 (*p* = 0.94) *n* = 181	−0.008 (*p* = 0.92) *n* = 180	−0.032 (*p* = 0.67) *n* = 180	−0.015 (*p* = 0.845) *n* = 182	0.005 (*p* = 0.95) *n* = 182	0.028 (*p* = 0.71) *n* = 181

Note: Spearman’s rank correlation was employed as the statistical method. 4βHC 4β-hydroxycholesterol; SBP systolic blood pressure; DBP diastolic blood pressure; BMI body mass index; MAP mean arterial pressure. We considered *p* < 0.05 statistically significant.

**Table 3.** Correlations of 4βHC and maternal and perinatal characteristics.

	Maternal BMI at the start of pregnancy	Maternal BMI at term	Maternal weight gain	Birth weight of the child	Weight of the placenta	Gestational age at the time of sampling	Maternal age at delivery
Normal pregnancies 4βHC	−0.135 (*p* = 0.42) *n* = 38	−0.119 (*p* = 0.48) *n* = 38	0.129 (*p* = 0.44) *n* = 38	0.202 (*p* = 0.22) *n* = 38	0.151 (*p* = 0.37) *n* = 37	0.069 (*p* = 0.76) *n* = 22	0.246 (*p* = 0.14) *n* = 38
Pre-eclampsia 4βHC	−0.205 **(*p* = 0.014)** *n* = 144	−0.230 **(*p* = 0.006)** *n* = 144	−0.033 (*p* = 0.69) *n* = 144	0.017 (*p* = 0.84) *n* = 144	0.031 (*p* = 0.72) *n* = 138	−0.138 (*p* = 0.27) *n* = 66	0.158 (*p* = 0.06) *n* = 144
All pregnancies 4βHC	−0.196 **(*p* = 0.008)** *n* = 182	−0.217 **(*p* = 0.003)** *n* = 182	−0.004 (*p* = 0.96) *n* = 182	0.026 (*p* = 0.723) *n* = 182	0.057 (*p* = 0.45) *n* = 175	−0.258 **(*p* = 0.015)** *n* = 88	0.172 (***p* = 0.020)** *n* = 182

Note: Spearman’s rank correlation was employed as the statistical method. 4βHC 4β-hydroxycholesterol; SBP systolic blood pressure; DBP diastolic blood pressure; BMI body mass index; MAP mean arterial pressure. We considered *p* < 0.05 statistically significant.

### Genetic associations in FinnGen

Significant associations between genetic variants located within or near the LXRα and LXRβ-encoding genes and hypertensive complications of pregnancy were not found. Further details can be seen in Supplementary Figures 1 and 2.

### Placental gene expression analysis between preeclamptic and normal pregnancy samples

**Figure 2.**
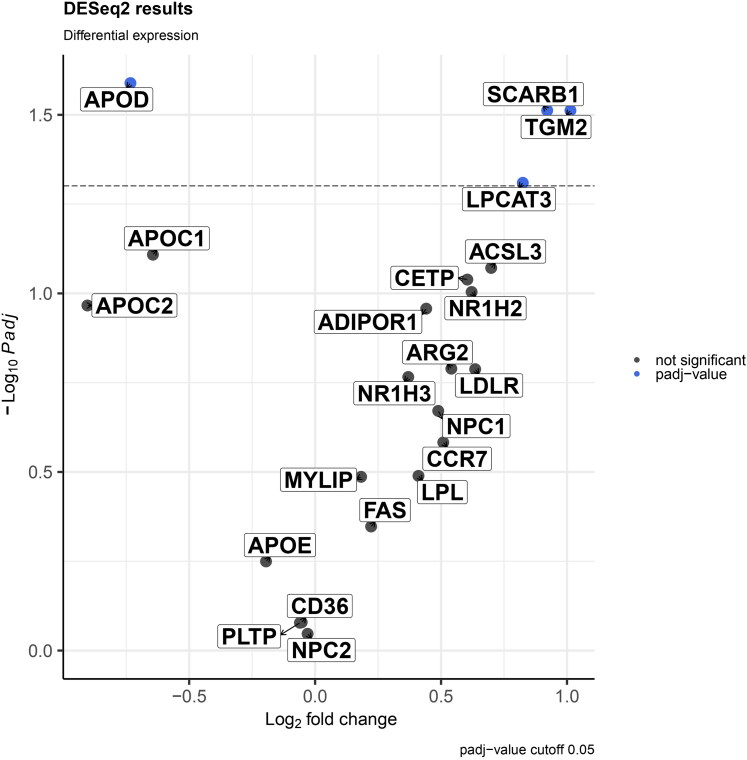
Differentially expressed genes in the LXR pathway in PE versus NP. Shown are significantly differentially expressed LXR genes (blue dots, padj < 0.05, DESeq2 Wald test) and non-significant genes (grey dots) in PE versus NP. The dashed line represents the significance threshold (padj = 0.05).

**Figure 3.**
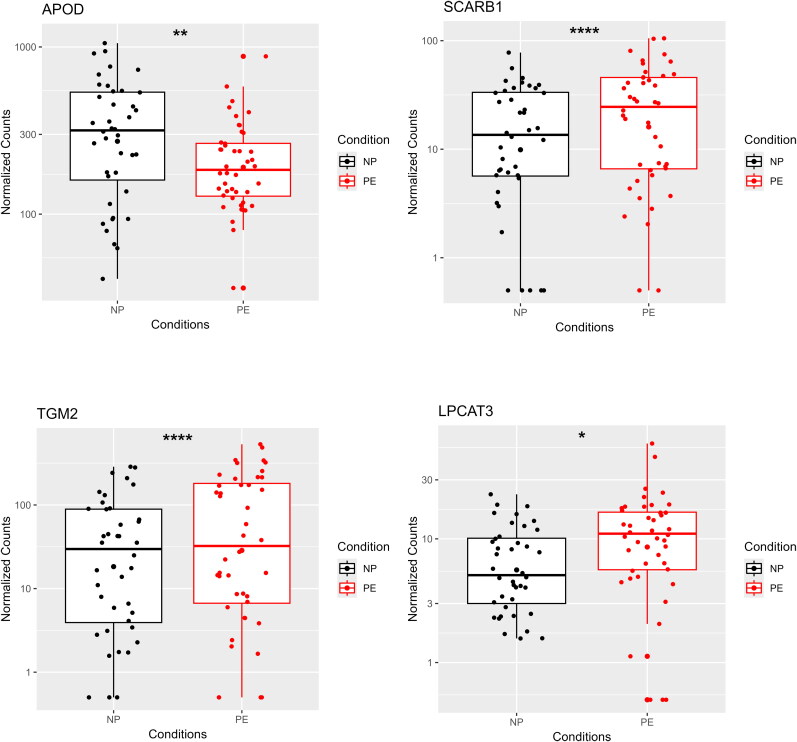
Differential expression of APOD, SCARB1, TGM2, and LPCAT3 between PE and NP conditions. Shown are the expression levels of APOD, SCARB1, TGM2, and LPCAT3, which were significantly different (padj < 0.05) between PE and NP conditions. Asterisks indicate statistical significance (*padj < 0.05, **padj < 0.01, ***padj < 0.001, ****padj < 0.0001).

Some significant differences in gene expression levels between the preeclampsia and normal pregnancy conditions were found for the genes known to be regulated *via* LXRs. APOD expression levels were lower on PE pregnancies compared to normal pregnancies (*p* < 0.01). SCARB1, TGM2 and LPCAT3 had higher expression levels in PE pregnancies compared to normal pregnancies (*p* < 0.0001, *p* < 0.0001 and *p* < 0.05). These findings are illustrated in Figures 2, 3, and Supplementary Figure 3.

### Correlation between placental expression and plasma oxysterols

LXR target genes that demonstrated significant correlation with oxysterols in normal pregnancies were identified. LPL exhibited a significant correlation with both 4αHC and 4βHC (*p* = 0.01 and *p* < 0.001 respectively). APOC1 exhibited a significant correlation with both 4αHC and 4βHC (*p* = 0.01 and *p* = 0.02, respectively). APOD exhibited a significant correlation with both 4αHC and 4βHC (*p* = 0.04 and *p* = 0.05, respectively). Additionally, the gene CD36 was found to correlate with 4αHC (*p* = 0.03). These findings are illustrated in Supplementary Figure 4.

Additionally, LXR target genes that exhibited significant differences in correlation with oxysterols between normal and PE pregnancies were identified. LPL displayed differential correlations with both 4αHC and 4βHC between the groups (*p* = 0.011 and *p* = 0.004, respectively). Similarly, APOC1 showed differential correlations with 4αHC and 4βHC between the groups (*p* = 0.007 and *p* = 0.009, respectively). APOD demonstrated significant differential correlations with both 4αHC and 4βHC between the groups (*p* = 0.013 and *p* = 0.011, respectively). Furthermore, CD36 was found to correlate differently with 4αHC between the groups (*p* = 0.019). FAS and NPC2 genes had significant differences in correlation with 4αHC between the groups (*p* = 0.021 and *p* = 0.040, respectively), but did not correlate inside the groups.

## Discussion

In this study, we investigated the associations between plasma 4βHC and BP as well as PE and maternal and perinatal characteristics in FINNPEC cohort. Despite our previous findings on a proposed new BP-regulating circuit, a connection between 4βHC and BP in pregnant women was not found. Also, no association between plasma 4βHC and pregnancy outcomes was detected. Thus, during the 3^rd^ trimester, plasma 4βHC is unlikely to regulate BP or pregnancy outcomes. In addition, we explored if the variants in genes encoding LXRα and LXRβ are associated with hypertensive pregnancy complications in FinnGen. This genetic approach provided evidence that the LXRs have no major role in PE and the regulation of BP in pregnancy.

To further explore the role of LXR pathway in PE, the expression of LXR target genes was analysed in placenta of PE and normal pregnancies. Interestingly, apolipoprotein D (APOD), transglutaminase 2 (TGM2), scavenger receptor class B member 1 (SCARB1) and lysophosphatidylcholine acyltransferase 3 (LPCAT3) had significant difference between the PE and normal pregnancies. Previous investigations have found that APOD gene expression is upregulated through LXR activation [44,45]. In this study, normal pregnancies demonstrated higher expression of placental APOD than PE pregnancies, a new observation. APOD is best recognised as a lipocalin that is involved in lipid transportation and defence mechanisms in oxidative stress [46]. APOD levels are altered in pregnancy conditions including gestational weight gain, gestational diabetes, and placenta accreta [47–49]. It can be speculated that lower APOD expression could predispose to PE as it may serve as a defence mechanism against oxidative stress occurring in PE in a similar way as in gestational diabetes [49].

TGM2 had higher placental expression level in PE pregnancies than in normal pregnancies. TGM2 expression has been found to be upregulated through LXR agonists [50]. It has been hypothesized that TGM2 stabilizes cytoskeletal particles in the syncytial microvillous membrane [51]. There is a reported difference in expression of cytoskeletal proteins in PE and normal pregnancies [52]. In similar fashion as TGM2, SCARB1 expression was increased in PE pregnancies. SCARB1 is not a direct target of LXR but it regulates transcriptionally PDZK1 which is known to stabilize SCARB1 protein [53]. Previous investigation found that high-density lipoprotein (HDL) receptor SCARB1 is a key element in materno-foetal cholesterol transport [54]. Also, PE pregnancies expressed more placental LPCAT3 gene than normal pregnancies. Earlier LPCAT3 has been found to be LXR target gene [55,56]. LPCAT3 is believed to play a part in regulating lipid levels in placental cells [57]. Thus, the LXR targets differentially regulated in PE are predominantly involved in the regulation of lipid metabolism.

The placental expression of certain LXR target genes was found to correlate with plasma oxysterols. Notably, APOD correlated positively with both 4αHC and 4βHC in normal pregnancies but not in PE pregnancies. Additionally, lipoprotein lipase (LPL) demonstrated a negative correlation with 4αHC and 4βHC in normal pregnancies but again not in PE. In contrast, apolipoprotein C-1 (APOC1) correlated positively with 4αHC and 4βHC in normal pregnancies. Furthermore, cluster of differentiation 36 (CD36) correlated positively with 4αHC in normal pregnancies. APOD, LPL, APOC1 and CD36 had significant differences in correlation between normal and PE pregnancies, which aligns logically with their significant correlations in normal pregnancies but not PE pregnancies. LPL, APOC1 and CD36 are all identified as target genes of LXR [58–60]. However, the finding that both 4αHC and 4βHC (or 4αHC alone) correlated with the placental expression levels argues against the role of 4βHC and LXR-mediated regulation of these genes as 4αHC is not an agonist for LXR.

Even if the 4βHC-LXR pathway does not seem to have an effect on gestational BP regulation in 3^rd^ trimester, there are still questions about the role of LXRs during pregnancy. There are some studies that have unveiled new aspects of how LXR activation could affect pregnancy outcomes. The main hypothesis revolves around pathophysiological development in pregnancy and the placenta when there is an absence of LXR activation [61]. The placenta acts as a barrier that regulates the flow of nutrients from the mother to the foetus, including cholesterol that serves as a precursor for many important hormones and cell structures, which are important for prenatal development [62]. LXR activation is important in early placental development and function as a lack of cholesterol and oxysterols, oxygenated derivatives of cholesterol, have been shown to inhibit trophoblast survival through the oxysterol-LXR pathway [63]. This study investigated the association of LXR and pregnancy outcomes using 4βHC levels as a marker for LXR agonism but due to sample availability limited to 3rd trimester, the role of 4βHC in BP regulation and PE at earlier trimesters requires further study.

Evaluating LXR activation through only one ligand is not ideal as there are multiple possible oxysterol ligands that may activate LXRs [15]. The same applies to the effects of oxysterol-induced alterations during gestation, as 4αHC and 4βHC only represent a small portion of the diverse oxysterol family. There are studies that have investigated the independent role of oxysterols in pregnancy and their proportions in plasma/serum compared to non-pregnant individuals. Nearly a decade ago, it was discovered that serum levels of 7α-hydroxycholesterol, 7β-hydroxycholesterol (7βHC), 4βHC, 20α-hydroxycholesterol, 24S-hydroxycholesterol and 27-hydroxycholesterol (27HC) were elevated in pregnancy [24]. Since then, 27HC has been found to upregulate extramedullary haematopoiesis during pregnancy in mice through hematopoietic stem cell mobilization [64]. One study found that 7βHC has an independent inflammatory-promoting effect on placental cells [17]. However, the genetic approach utilizing FinnGen data in this study found no evidence for the role of LXRα and LXRβ in PE, suggesting that LXRs may not have significance in placental dysfunction and PE.

Results regarding differences between PE and control groups in BP, the placental weight, child weight, and gestational age are typical for PE [65]. Smaller placenta in PE patients could be a direct outcome of shorter gestation, but the median difference of 122 g seems to be quite excessive with a 3-week median difference in delivery. This might apply to some extent to the birth weight of the children. However, the substantial difference between PE and control groups in the birth weight (831 g median difference) and the great variability of the birth weight of the children in the PE group (1166 g IQR) are most likely the results of PE hampering the function of placenta and not explained by the shorter gestation.

Previous results [25] indicating elevated plasma 4βHC during pregnancy (1.4-, 1.9, and 2.3-fold higher in the 1st, 2nd, and 3rd trimesters, respectively) were confirmed as the median 4βHC plasma concentration of 46 ng/ml detected here in 3^rd^ trimester is about 2.9-fold higher compared to healthy non-pregnant female volunteers in our previous study [20]. However, no significant differences were observed in the plasma concentrations of plasma 4βHC and 4αHC between the PE and control groups, although a previous study reported 1.6-fold higher levels of serum 4βHC in patients with PE than in normal pregnancies [24]. Previous studies have used 15 ng/ml as a maximum limit for plasma 4αHC in non-pregnant individuals [66]. The 4αHC levels were considerably higher in this study but this is not necessarily abnormal as a previous study reported elevated 4αHC levels during pregnancy [43].

The negative correlation between plasma 4βHC and maternal BMI was in line with previous studies performed with non-pregnant individuals [67,68]. Maternal age correlated positively with 4βHC, which is a novel observation. It should be noted that previous studies have not demonstrated a significant association between plasma 4βHC levels and age in general adult cohorts [69,70].

Based on previous studies, it is reasonable to speculate that oxysterols may have a role in changes affecting circulation and placental development in pregnancy and PE. Furthermore, it can be hypothesized that the various oxysterols may have both aggregate and individual LXR-mediated impacts on the vascular system but also involve non-LXR-mediated effects. These effects may also vary, in manners specific to individual oxysterols, according to the phases of pregnancy. More research, for example with placental lipidomic profiling approach [71], is needed to determine the totality of factors that affect oxysterol- and LXR-mediated vascular changes in the gestational period. We believe that identifying the underlying factors and mechanisms behind PE and other pregnancy complications is important for primary prevention in risk groups for placental dysfunction.

## Supplementary Material

Supplemental Material

## Data Availability

The data that support the findings of this study are available on request from the corresponding author, LK. The data are not publicly available due to that some access restrictions apply to the FINNPEC data. The researchers interested in using the FINNPEC data must obtain approval from the FINNPEC Board (steering committee). The researchers using the data are required to follow the terms of a number of clauses designed to ensure the protection of privacy and compliance with relevant Finnish laws. Data requests may be subject to further review by the Ethics Committee and may also be subject to individual participant consent. The FinnGen data that support the findings of this study are available from FinnGen at www.finngen.fi.
