# Supplementary material for: Role of oxysterol 4β-hydroxycholesterol and liver X receptor alleles in pre-eclampsia
Source: Ann Med. 2025 Apr 29;57(1):2495763. doi: 10.1080/07853890.2025.2495763 (PMC12042236; doi:10.1080/07853890.2025.2495763)
Supplement: Supplemental Material [file IANN_A_2495763_SM9417.zip › Suppl/Clean copy - Supplements.docx]

# Supplements

The FinnGen study is approved by Finnish Institute for Health and Welfare (permit numbers: THL/2031/6.02.00/2017, THL/1101/5.05.00/2017, THL/341/6.02.00/2018, THL/2222/6.02.00/2018, THL/283/6.02.00/2019, THL/1721/5.05.00/2019 and THL/1524/5.05.00/2020), Digital and population data service agency (permit numbers: VRK43431/2017-3, VRK/6909/2018-3, VRK/4415/2019-3), the Social Insurance Institution (permit numbers: KELA 58/522/2017, KELA 131/522/2018, KELA 70/522/2019, KELA 98/522/2019, KELA 134/522/2019, KELA 138/522/2019, KELA 2/522/2020, KELA 16/522/2020), Findata permit numbers THL/2364/14.02/2020, THL/4055/14.06.00/2020,,THL/3433/14.06.00/2020, THL/4432/14.06/2020, THL/5189/14.06/2020, THL/5894/14.06.00/2020, THL/6619/14.06.00/2020, THL/209/14.06.00/2021, THL/688/14.06.00/2021, THL/1284/14.06.00/2021, THL/1965/14.06.00/2021, THL/5546/14.02.00/2020, THL/2658/14.06.00/2021, THL/4235/14.06.00/202, Statistics Finland (permit numbers: TK-53-1041-17 and TK/143/07.03.00/2020 (earlier TK-53-90-20) TK/1735/07.03.00/2021, TK/3112/07.03.00/2021) and Finnish Registry for Kidney Diseases permission/extract from the meeting minutes on 4th July 2019. The Biobank Access Decisions for FinnGen samples and data utilized in FinnGen Data Freeze 9 include: THL Biobank BB2017_55, BB2017_111, BB2018_19, BB_2018_34, BB_2018_67, BB2018_71, BB2019_7, BB2019_8, BB2019_26, BB2020_1, Finnish Red Cross Blood Service Biobank 7.12.2017, Helsinki Biobank HUS/359/2017, HUS/248/2020, Auria Biobank AB17-5154 and amendment #1 (August 17 2020), AB20-5926 and amendment #1 (April 23 2020) and it´s modification (Sep 22 2021), Biobank Borealis of Northern Finland_2017_1013, Biobank of Eastern Finland 1186/2018 and amendment 22 § /2020, Finnish Clinical Biobank Tampere MH0004 and amendments (21.02.2020 & 06.10.2020), Central Finland Biobank 1-2017, and Terveystalo Biobank STB 2018001 and amendment 25th Aug 2020.

# FinnGen authors

| **Full Name** | **Affiliation** |
| --- | --- |
| Aarno Palotie | Institute for Molecular Medicine Finland (FIMM), HiLIFE, University of Helsinki, Helsinki, Finland; Broad Institute of MIT and Harvard; Massachusetts General Hospital |
| Mark Daly | Institute for Molecular Medicine Finland (FIMM), HiLIFE, University of Helsinki, Helsinki, Finland; Broad Institute of MIT and Harvard; Massachusetts General Hospital |
| Bridget Riley-Gills | Abbvie, Chicago, IL, United States |
| Howard Jacob | Abbvie, Chicago, IL, United States |
| Dirk Paul | Astra Zeneca, Cambridge, United Kingdom |
| Slavé Petrovski | Astra Zeneca, Cambridge, United Kingdom |
| Chia-Yen Chen | Biogen, Cambridge, MA, United States |
| Sally John | Biogen, Cambridge, MA, United States |
| George Okafo | Boehringer Ingelheim, Ingelheim am Rhein, Germany |
| Robert Plenge | Bristol Myers Squibb, New York, NY, United States |
| Joseph Maranville | Bristol Myers Squibb, New York, NY, United States |
| Mark McCarthy | Genentech, San Francisco, CA, United States |
| Rion Pendergrass | Genentech, San Francisco, CA, United States |
| Margaret G. Ehm | GlaxoSmithKline, Collegeville, PA, United States |
| Kirsi Auro | GlaxoSmithKline, Espoo, Finland |
| Simonne Longerich | Merck, Kenilworth, NJ, United States |
| Anders Mälarstig | Pfizer, New York, NY, United States |
| Anna Vlahiotis | Pfizer, New York, NY, United States |
| Katherine Klinger | Translational Sciences, Sanofi R&D, Framingham, MA, USA |
| Clement Chatelain | Translational Sciences, Sanofi R&D, Framingham, MA, USA |
| Matthias Gossel | Translational Sciences, Sanofi R&D, Framingham, MA, USA |
| Karol Estrada | Maze Therapeutics, San Francisco, CA, United States |
| Robert Graham | Maze Therapeutics, San Francisco, CA, United States |
| Dawn Waterworth | Janssen Research & Development, LLC, Spring House, PA, United States |
| Chris O´Donnell | Novartis Institutes for BioMedical Research, Cambridge, MA, United States |
| Nicole Renaud | Novartis Institutes for BioMedical Research, Cambridge, MA, United States |
| Tomi P. Mäkelä | HiLIFE, University of Helsinki, Finland, Finland |
| Jaakko Kaprio | Institute for Molecular Medicine Finland (FIMM), HiLIFE, University of Helsinki, Helsinki, Finland |
| Minna Ruddock | Arctic biobank / University of Oulu |
| Petri Virolainen | Auria Biobank / University of Turku / Hospital District of Southwest Finland, Turku, Finland |
| Antti Hakanen | Auria Biobank / University of Turku / Hospital District of Southwest Finland, Turku, Finland |
| Terhi Kilpi | THL Biobank / Finnish Institute for Health and Welfare (THL), Helsinki, Finland |
| Markus Perola | THL Biobank / Finnish Institute for Health and Welfare (THL), Helsinki, Finland |
| Jukka Partanen | Finnish Red Cross Blood Service / Finnish Hematology Registry and Clinical Biobank, Helsinki, Finland |
| Taneli Raivio | Helsinki Biobank / Helsinki University and Hospital District of Helsinki and Uusimaa, Helsinki |
| Jani Tikkanen | Northern Finland Biobank Borealis / University of Oulu / Northern Ostrobothnia Hospital District, Oulu, Finland |
| Raisa Serpi | Northern Finland Biobank Borealis / University of Oulu / Northern Ostrobothnia Hospital District, Oulu, Finland |
| Tarja Laitinen | Finnish Clinical Biobank Tampere / University of Tampere / Pirkanmaa Hospital District, Tampere, Finland |
| Veli-Matti Kosma | Biobank of Eastern Finland / University of Eastern Finland / Northern Savo Hospital District, Kuopio, Finland |
| Jari Laukkanen | Central Finland Biobank / University of Jyväskylä / Central Finland Health Care District, Jyväskylä, Finland |
| Marco Hautalahti | FINBB - Finnish biobank cooperative |
| Outi Tuovila | Business Finland, Helsinki, Finland |
| Raimo Pakkanen | Business Finland, Helsinki, Finland |
| Jeffrey Waring | Abbvie, Chicago, IL, United States |
| Bridget Riley-Gillis | Abbvie, Chicago, IL, United States |
| Fedik Rahimov | Abbvie, Chicago, IL, United States |
| Ioanna Tachmazidou | Astra Zeneca, Cambridge, United Kingdom |
| Chia-Yen Chen | Biogen, Cambridge, MA, United States |
| Zhihao Ding | Boehringer Ingelheim, Ingelheim am Rhein, Germany |
| Marc Jung | Boehringer Ingelheim, Ingelheim am Rhein, Germany |
| Hanati Tuoken | Boehringer Ingelheim, Ingelheim am Rhein, Germany |
| Shameek Biswas | Bristol Myers Squibb, New York, NY, United States |
| Rion Pendergrass | Genentech, San Francisco, CA, United States |
| Margaret G. Ehm | GlaxoSmithKline, Collegeville, PA, United States |
| David Pulford | GlaxoSmithKline, Stevenage, United Kingdom |
| Neha Raghavan | Merck, Kenilworth, NJ, United States |
| Adriana Huertas-Vazquez | Merck, Kenilworth, NJ, United States |
| Jae-Hoon Sul | Merck, Kenilworth, NJ, United States |
| Anders Mälarstig | Pfizer, New York, NY, United States |
| Xinli Hu | Pfizer, New York, NY, United States |
| Åsa Hedman | Pfizer, New York, NY, United States |
| Katherine Klinger | Translational Sciences, Sanofi R&D, Framingham, MA, USA |
| Robert Graham | Maze Therapeutics, San Francisco, CA, United States |
| Dawn Waterworth | Janssen Research & Development, LLC, Spring House, PA, United States |
| Nicole Renaud | Novartis Institutes for BioMedical Research, Cambridge, MA, United States |
| Ma´en Obeidat | Novartis Institutes for BioMedical Research, Cambridge, MA, United States |
| Jonathan Chung | Novartis Institutes for BioMedical Research, Cambridge, MA, United States |
| Jonas Zierer | Novartis Institutes for BioMedical Research, Cambridge, MA, United States |
| Mari Niemi | Novartis Institutes for BioMedical Research, Cambridge, MA, United States |
| Samuli Ripatti | Institute for Molecular Medicine Finland (FIMM), HiLIFE, University of Helsinki, Helsinki, Finland |
| Johanna Schleutker | Auria Biobank / Univ. of Turku / Hospital District of Southwest Finland, Turku, Finland |
| Markus Perola | THL Biobank / Finnish Institute for Health and Welfare (THL), Helsinki, Finland |
| Mikko Arvas | Finnish Red Cross Blood Service / Finnish Hematology Registry and Clinical Biobank, Helsinki, Finland |
| Olli Carpén | Helsinki Biobank / Helsinki University and Hospital District of Helsinki and Uusimaa, Helsinki |
| Reetta Hinttala | Northern Finland Biobank Borealis / University of Oulu / Northern Ostrobothnia Hospital District, Oulu, Finland |
| Johannes Kettunen | Northern Finland Biobank Borealis / University of Oulu / Northern Ostrobothnia Hospital District, Oulu, Finland |
| Arto Mannermaa | Biobank of Eastern Finland / University of Eastern Finland / Northern Savo Hospital District, Kuopio, Finland |
| Katriina Aalto-Setälä | Faculty of Medicine and Health Technology, Tampere University, Tampere, Finland |
| Mika Kähönen | Finnish Clinical Biobank Tampere / University of Tampere / Pirkanmaa Hospital District, Tampere, Finland |
| Jari Laukkanen | Central Finland Biobank / University of Jyväskylä / Central Finland Health Care District, Jyväskylä, Finland |
| Johanna Mäkelä | FINBB - Finnish biobank cooperative |
| Reetta Kälviäinen | Northern Savo Hospital District, Kuopio, Finland |
| Valtteri Julkunen | Northern Savo Hospital District, Kuopio, Finland |
| Hilkka Soininen | Northern Savo Hospital District, Kuopio, Finland |
| Anne Remes | Northern Ostrobothnia Hospital District, Oulu, Finland |
| Mikko Hiltunen | University of Eastern Finland, Kuopio, Finland |
| Jukka Peltola | Pirkanmaa Hospital District, Tampere, Finland |
| Minna Raivio | Hospital District of Helsinki and Uusimaa, Helsinki, Finland |
| Pentti Tienari | Hospital District of Helsinki and Uusimaa, Helsinki, Finland |
| Juha Rinne | Hospital District of Southwest Finland, Turku, Finland |
| Roosa Kallionpää | Hospital District of Southwest Finland, Turku, Finland |
| Juulia Partanen | Institute for Molecular Medicine Finland, HiLIFE, University of Helsinki, Finland |
| Adam Ziemann | Abbvie, Chicago, IL, United States |
| Nizar Smaoui | Abbvie, Chicago, IL, United States |
| Anne Lehtonen | Abbvie, Chicago, IL, United States |
| Susan Eaton | Biogen, Cambridge, MA, United States |
| Heiko Runz | Biogen, Cambridge, MA, United States |
| Sanni Lahdenperä | Biogen, Cambridge, MA, United States |
| Shameek Biswas | Bristol Myers Squibb, New York, NY, United States |
| Natalie Bowers | Genentech, San Francisco, CA, United States |
| Edmond Teng | Genentech, San Francisco, CA, United States |
| Rion Pendergrass | Genentech, San Francisco, CA, United States |
| Fanli Xu | GlaxoSmithKline, Brentford, United Kingdom |
| David Pulford | GlaxoSmithKline, Stevenage, United Kingdom |
| Kirsi Auro | GlaxoSmithKline, Espoo, Finland |
| Laura Addis | GlaxoSmithKline, Brentford, United Kingdom |
| John Eicher | GlaxoSmithKline, Brentford, United Kingdom |
| Qingqin S Li | Janssen Research & Development, LLC, Titusville, NJ 08560, United States |
| Karen He | Janssen Research & Development, LLC, Spring House, PA, United States |
| Ekaterina Khramtsova | Janssen Research & Development, LLC, Spring House, PA, United States |
| Neha Raghavan | Merck, Kenilworth, NJ, United States |
| Martti Färkkilä | Hospital District of Helsinki and Uusimaa, Helsinki, Finland |
| Jukka Koskela | Hospital District of Helsinki and Uusimaa, Helsinki, Finland |
| Sampsa Pikkarainen | Hospital District of Helsinki and Uusimaa, Helsinki, Finland |
| Airi Jussila | Pirkanmaa Hospital District, Tampere, Finland |
| Katri Kaukinen | Pirkanmaa Hospital District, Tampere, Finland |
| Timo Blomster | Northern Ostrobothnia Hospital District, Oulu, Finland |
| Mikko Kiviniemi | Northern Savo Hospital District, Kuopio, Finland |
| Markku Voutilainen | Hospital District of Southwest Finland, Turku, Finland |
| Mark Daly | Institute for Molecular Medicine, Finland (FIMM), HiLIFE, University of Helsinki, Helsinki, Finland; Broad Institute of MIT and Harvard; Massachusetts General Hospital |
| Jeffrey Waring | Abbvie, Chicago, IL, United States |
| Nizar Smaoui | Abbvie, Chicago, IL, United States |
| Fedik Rahimov | Abbvie, Chicago, IL, United States |
| Anne Lehtonen | Abbvie, Chicago, IL, United States |
| Tim Lu | Genentech, San Francisco, CA, United States |
| Natalie Bowers | Genentech, San Francisco, CA, United States |
| Rion Pendergrass | Genentech, San Francisco, CA, United States |
| Linda McCarthy | GlaxoSmithKline, Brentford, United Kingdom |
| Amy Hart | Janssen Research & Development, LLC, Spring House, PA, United States |
| Meijian Guan | Janssen Research & Development, LLC, Spring House, PA, United States |
| Jason Miller | Merck, Kenilworth, NJ, United States |
| Kirsi Kalpala | Pfizer, New York, NY, United States |
| Melissa Miller | Pfizer, New York, NY, United States |
| Xinli Hu | Pfizer, New York, NY, United States |
| Kari Eklund | Hospital District of Helsinki and Uusimaa, Helsinki, Finland |
| Antti Palomäki | Hospital District of Southwest Finland, Turku, Finland |
| Pia Isomäki | Pirkanmaa Hospital District, Tampere, Finland |
| Laura Pirilä | Hospital District of Southwest Finland, Turku, Finland |
| Oili Kaipiainen-Seppänen | Northern Savo Hospital District, Kuopio, Finland |
| Johanna Huhtakangas | Northern Ostrobothnia Hospital District, Oulu, Finland |
| Nina Mars | Institute for Molecular Medicine Finland (FIMM), HiLIFE, University of Helsinki, Helsinki, Finland |
| Jeffrey Waring | Abbvie, Chicago, IL, United States |
| Fedik Rahimov | Abbvie, Chicago, IL, United States |
| Apinya Lertratanakul | Abbvie, Chicago, IL, United States |
| Nizar Smaoui | Abbvie, Chicago, IL, United States |
| Anne Lehtonen | Abbvie, Chicago, IL, United States |
| Coralie Viollet | AstraZeneca, Cambridge, United Kingdom |
| Marla Hochfeld | Bristol Myers Squibb, New York, NY, United States |
| Natalie Bowers | Genentech, San Francisco, CA, United States |
| Rion Pendergrass | Genentech, San Francisco, CA, United States |
| Jorge Esparza Gordillo | GlaxoSmithKline, Brentford, United Kingdom |
| Kirsi Auro | GlaxoSmithKline, Espoo, Finland |
| Dawn Waterworth | Janssen Research & Development, LLC, Spring House, PA, United States |
| Fabiana Farias | Merck, Kenilworth, NJ, United States |
| Kirsi Kalpala | Pfizer, New York, NY, United States |
| Nan Bing | Pfizer, New York, NY, United States |
| Xinli Hu | Pfizer, New York, NY, United States |
| Tarja Laitinen | Pirkanmaa Hospital District, Tampere, Finland |
| Margit Pelkonen | Northern Savo Hospital District, Kuopio, Finland |
| Paula Kauppi | Hospital District of Helsinki and Uusimaa, Helsinki, Finland |
| Hannu Kankaanranta | University of Gothenburg, Gothenburg, Sweden/ Seinäjoki Central Hospital, Seinäjoki, Finland/ Tampere University, Tampere, Finland |
| Terttu Harju | Northern Ostrobothnia Hospital District, Oulu, Finland |
| Riitta Lahesmaa | Hospital District of Southwest Finland, Turku, Finland |
| Nizar Smaoui | Abbvie, Chicago, IL, United States |
| Coralie Viollet | AstraZeneca, Cambridge, United Kingdom |
| Susan Eaton | Biogen, Cambridge, MA, United States |
| Hubert Chen | Genentech, San Francisco, CA, United States |
| Rion Pendergrass | Genentech, San Francisco, CA, United States |
| Natalie Bowers | Genentech, San Francisco, CA, United States |
| Joanna Betts | GlaxoSmithKline, Brentford, United Kingdom |
| Kirsi Auro | GlaxoSmithKline, Espoo, Finland |
| Rajashree Mishra | GlaxoSmithKline, Brentford, United Kingdom |
| Majd Mouded | Novartis, Basel, Switzerland |
| Debby Ngo | Novartis, Basel, Switzerland |
| Teemu Niiranen | Finnish Institute for Health and Welfare (THL), Helsinki, Finland |
| Felix Vaura | Finnish Institute for Health and Welfare (THL), Helsinki, Finland |
| Veikko Salomaa | Finnish Institute for Health and Welfare (THL), Helsinki, Finland |
| Kaj Metsärinne | Hospital District of Southwest Finland, Turku, Finland |
| Jenni Aittokallio | Hospital District of Southwest Finland, Turku, Finland |
| Mika Kähönen | Pirkanmaa Hospital District, Tampere, Finland |
| Jussi Hernesniemi | Pirkanmaa Hospital District, Tampere, Finland |
| Daniel Gordin | Hospital District of Helsinki and Uusimaa, Helsinki, Finland |
| Juha Sinisalo | Hospital District of Helsinki and Uusimaa, Helsinki, Finland |
| Marja-Riitta Taskinen | Hospital District of Helsinki and Uusimaa, Helsinki, Finland |
| Tiinamaija Tuomi | Hospital District of Helsinki and Uusimaa, Helsinki, Finland |
| Timo Hiltunen | Hospital District of Helsinki and Uusimaa, Helsinki, Finland |
| Jari Laukkanen | Central Finland Health Care District, Jyväskylä, Finland |
| Amanda Elliott | Institute for Molecular Medicine Finland (FIMM), HiLIFE, University of Helsinki, Helsinki, Finland; Broad Institute, Cambridge, MA, USA and Massachusetts General Hospital, Boston, MA, USA |
| Mary Pat Reeve | Institute for Molecular Medicine Finland (FIMM), HiLIFE, University of Helsinki, Helsinki, Finland |
| Sanni Ruotsalainen | Institute for Molecular Medicine Finland (FIMM), HiLIFE, University of Helsinki, Helsinki, Finland |
| Dirk Paul | Astra Zeneca, Cambridge, United Kingdom |
| Natalie Bowers | Genentech, San Francisco, CA, United States |
| Rion Pendergrass | Genentech, San Francisco, CA, United States |
| Audrey Chu | GlaxoSmithKline, Brentford, United Kingdom |
| Kirsi Auro | GlaxoSmithKline, Espoo, Finland |
| Dermot Reilly | Janssen Research & Development, LLC, Boston, MA, United States |
| Mike Mendelson | Novartis, Boston, MA, United States |
| Jaakko Parkkinen | Pfizer, New York, NY, United States |
| Melissa Miller | Pfizer, New York, NY, United States |
| Tuomo Meretoja | Department of Breast Surgery, Helsinki University Hospital Comprehensive Cancer Center and University of Helsinki, Helsinki, Finland |
| Heikki Joensuu | Department of Oncology, Helsinki University Hospital Comprehensive Cancer Center and University of Helsinki, Helsinki, Finland |
| Olli Carpén | Hospital District of Helsinki and Uusimaa, Helsinki, Finland |
| Johanna Mattson | Hospital District of Helsinki and Uusimaa, Helsinki, Finland |
| Eveliina Salminen | Hospital District of Helsinki and Uusimaa, Helsinki, Finland |
| Annika Auranen | Pirkanmaa Hospital District , Tampere, Finland |
| Peeter Karihtala | Department of Oncology, Helsinki University Hospital Comprehensive Cancer Center and University of Helsinki, Helsinki, Finland |
| Päivi Auvinen | Northern Savo Hospital District, Kuopio, Finland |
| Klaus Elenius | Hospital District of Southwest Finland, Turku, Finland |
| Johanna Schleutker | Hospital District of Southwest Finland, Turku, Finland |
| Esa Pitkänen | Institute for Molecular Medicine Finland (FIMM), HiLIFE, University of Helsinki, Helsinki, Finland |
| Nina Mars | Institute for Molecular Medicine Finland (FIMM), HiLIFE, University of Helsinki, Helsinki, Finland |
| Mark Daly | Institute for Molecular Medicine Finland (FIMM), HiLIFE, University of Helsinki, Helsinki, Finland; Broad Institute of MIT and Harvard; Massachusetts General Hospital |
| Relja Popovic | Abbvie, Chicago, IL, United States |
| Jeffrey Waring | Abbvie, Chicago, IL, United States |
| Bridget Riley-Gillis | Abbvie, Chicago, IL, United States |
| Anne Lehtonen | Abbvie, Chicago, IL, United States |
| Margarete Fabre | AstraZeneca, Cambridge, United Kingdom |
| Jennifer Schutzman | Genentech, San Francisco, CA, United States |
| Natalie Bowers | Genentech, San Francisco, CA, United States |
| Rion Pendergrass | Genentech, San Francisco, CA, United States |
| Diptee Kulkarni | GlaxoSmithKline, Brentford, United Kingdom |
| Kirsi Auro | GlaxoSmithKline, Espoo, Finland |
| Alessandro Porello | Janssen Research & Development, LLC, Spring House, PA, United States |
| Andrey Loboda | Merck, Kenilworth, NJ, United States |
| Heli Lehtonen | Pfizer, New York, NY, United States |
| Stefan McDonough | Pfizer, New York, NY, United States |
| Sauli Vuoti | Janssen-Cilag Oy, Espoo, Finland |
| Kai Kaarniranta | Northern Savo Hospital District, Kuopio, Finland; Department of Molecular Genetics, University of Lodz, Lodz, Poland |
| Joni A Turunen | Helsinki University Hospital and University of Helsinki, Helsinki, Finland; Eye Genetics Group, Folkhälsan Research Center, Helsinki, Finland |
| Terhi Ollila | Hospital District of Helsinki and Uusimaa, Helsinki, Finland |
| Hannu Uusitalo | Pirkanmaa Hospital District, Tampere, Finland |
| Juha Karjalainen | Institute for Molecular Medicine Finland (FIMM), HiLIFE, University of Helsinki, Helsinki, Finland |
| Esa Pitkänen | Institute for Molecular Medicine Finland (FIMM), HiLIFE, University of Helsinki, Helsinki, Finland |
| Mengzhen Liu | Abbvie, Chicago, IL, United States |
| Heiko Runz | Biogen, Cambridge, MA, United States |
| Stephanie Loomis | Biogen, Cambridge, MA, United States |
| Erich Strauss | Genentech, San Francisco, CA, United States |
| Natalie Bowers | Genentech, San Francisco, CA, United States |
| Hao Chen | Genentech, San Francisco, CA, United States |
| Rion Pendergrass | Genentech, San Francisco, CA, United States |
| Kaisa Tasanen | Northern Ostrobothnia Hospital District, Oulu, Finland |
| Laura Huilaja | Northern Ostrobothnia Hospital District, Oulu, Finland |
| Katariina Hannula-Jouppi | Hospital District of Helsinki and Uusimaa, Helsinki, Finland |
| Teea Salmi | Pirkanmaa Hospital District, Tampere, Finland |
| Sirkku Peltonen | Hospital District of Southwest Finland, Turku, Finland |
| Leena Koulu | Hospital District of Southwest Finland, Turku, Finland |
| Nizar Smaoui | Abbvie, Chicago, IL, United States |
| Fedik Rahimov | Abbvie, Chicago, IL, United States |
| Anne Lehtonen | Abbvie, Chicago, IL, United States |
| David Choy | Genentech, San Francisco, CA, United States |
| Rion Pendergrass | Genentech, San Francisco, CA, United States |
| Dawn Waterworth | Janssen Research & Development, LLC, Spring House, PA, United States |
| Kirsi Kalpala | Pfizer, New York, NY, United States |
| Ying Wu | Pfizer, New York, NY, United States |
| Pirkko Pussinen | Hospital District of Helsinki and Uusimaa, Helsinki, Finland |
| Aino Salminen | Hospital District of Helsinki and Uusimaa, Helsinki, Finland |
| Tuula Salo | Hospital District of Helsinki and Uusimaa, Helsinki, Finland |
| David Rice | Hospital District of Helsinki and Uusimaa, Helsinki, Finland |
| Pekka Nieminen | Hospital District of Helsinki and Uusimaa, Helsinki, Finland |
| Ulla Palotie | Hospital District of Helsinki and Uusimaa, Helsinki, Finland |
| Maria Siponen | Northern Savo Hospital District, Kuopio, Finland |
| Liisa Suominen | Northern Savo Hospital District, Kuopio, Finland |
| Päivi Mäntylä | Northern Savo Hospital District, Kuopio, Finland |
| Ulvi Gursoy | Hospital District of Southwest Finland, Turku, Finland |
| Vuokko Anttonen | Northern Ostrobothnia Hospital District, Oulu, Finland |
| Kirsi Sipilä | Research Unit of Oral Health Sciences Faculty of Medicine, University of Oulu, Oulu, Finland; Medical Research Center, Oulu, Oulu University Hospital and University of Oulu, Oulu, Finland |
| Rion Pendergrass | Genentech, San Francisco, CA, United States |
| Hannele Laivuori | Institute for Molecular Medicine Finland (FIMM), HiLIFE, University of Helsinki, Helsinki, Finland |
| Venla Kurra | Pirkanmaa Hospital District, Tampere, Finland |
| Laura Kotaniemi-Talonen | Pirkanmaa Hospital District, Tampere, Finland |
| Oskari Heikinheimo | Hospital District of Helsinki and Uusimaa, Helsinki, Finland |
| Ilkka Kalliala | Hospital District of Helsinki and Uusimaa, Helsinki, Finland |
| Lauri Aaltonen | Hospital District of Helsinki and Uusimaa, Helsinki, Finland |
| Varpu Jokimaa | Hospital District of Southwest Finland, Turku, Finland |
| Johannes Kettunen | Northern Ostrobothnia Hospital District, Oulu, Finland |
| Marja Vääräsmäki | Northern Ostrobothnia Hospital District, Oulu, Finland |
| Outi Uimari | Northern Ostrobothnia Hospital District, Oulu, Finland |
| Laure Morin-Papunen | Northern Ostrobothnia Hospital District, Oulu, Finland |
| Maarit Niinimäki | Northern Ostrobothnia Hospital District, Oulu, Finland |
| Terhi Piltonen | Northern Ostrobothnia Hospital District, Oulu, Finland |
| Katja Kivinen | Institute for Molecular Medicine Finland (FIMM), HiLIFE, University of Helsinki, Helsinki, Finland |
| Elisabeth Widen | Institute for Molecular Medicine Finland (FIMM), HiLIFE, University of Helsinki, Helsinki, Finland |
| Taru Tukiainen | Institute for Molecular Medicine Finland (FIMM), HiLIFE, University of Helsinki, Helsinki, Finland |
| Mary Pat Reeve | Institute for Molecular Medicine Finland (FIMM), HiLIFE, University of Helsinki, Helsinki, Finland |
| Mark Daly | Institute for Molecular Medicine Finland (FIMM), HiLIFE, University of Helsinki, Helsinki, Finland; Broad Institute of MIT and Harvard; Massachusetts General Hospital |
| Niko Välimäki | University of Helsinki, Helsinki, Finland |
| Eija Laakkonen | University of Jyväskylä, Jyväskylä, Finland |
| Jaakko Tyrmi | University of Oulu, Oulu, Finland / University of Tampere, Tampere, Finland |
| Heidi Silven | University of Oulu, Oulu, Finland |
| Eeva Sliz | University of Oulu, Oulu, Finland |
| Riikka Arffman | University of Oulu, Oulu, Finland |
| Susanna Savukoski | University of Oulu, Oulu, Finland |
| Triin Laisk | Estonian biobank, Tartu, Estonia |
| Natalia Pujol | Estonian biobank, Tartu, Estonia |
| Mengzhen Liu | Abbvie, Chicago, IL, United States |
| Bridget Riley-Gillis | Abbvie, Chicago, IL, United States |
| Rion Pendergrass | Genentech, San Francisco, CA, United States |
| Janet Kumar | GlaxoSmithKline, Collegeville, PA, United States |
| Kirsi Auro | GlaxoSmithKline, Espoo, Finland |
| Iiris Hovatta | University of Helsinki, Finland |
| Chia-Yen Chen | Biogen, Cambridge, MA, United States |
| Erkki Isometsä | Hospital District of Helsinki and Uusimaa, Helsinki, Finland |
| Hanna Ollila | Institute for Molecular Medicine Finland (FIMM), HiLIFE, University of Helsinki, Helsinki, Finland |
| Jaana Suvisaari | Finnish Institute for Health and Welfare (THL), Helsinki, Finland |
| Antti Mäkitie | Department of Otorhinolaryngology - Head and Neck Surgery, University of Helsinki and Helsinki University Hospital, Helsinki, Finland |
| Argyro Bizaki-Vallaskangas | Pirkanmaa Hospital District, Tampere, Finland |
| Sanna Toppila-Salmi | University of Eastern Finland and Kuopio University Hospital, Department of Otorhinolaryngology, Kuopio, Finland and Department of Allergy, Helsinki University Hospital and University of Helsinki, Finland |
| Tytti Willberg | Hospital District of Southwest Finland, Turku, Finland |
| Elmo Saarentaus | Institute for Molecular Medicine Finland (FIMM), HiLIFE, University of Helsinki, Helsinki, Finland |
| Antti Aarnisalo | Hospital District of Helsinki and Uusimaa, Helsinki, Finland |
| Eveliina Salminen | Hospital District of Helsinki and Uusimaa, Helsinki, Finland |
| Elisa Rahikkala | Northern Ostrobothnia Hospital District, Oulu, Finland |
| Johannes Kettunen | Northern Ostrobothnia Hospital District, Oulu, Finland |
| Kristiina Aittomäki | Department of Medical Genetics, Helsinki University Central Hospital, Helsinki, Finland |
| Fredrik Åberg | Transplantation and Liver Surgery Clinic, Helsinki University Hospital, Helsinki University, Helsinki, Finland |
| Mitja Kurki | Institute for Molecular Medicine Finland (FIMM), HiLIFE, University of Helsinki, Helsinki, Finland; Broad Institute, Cambridge, MA, United States |
| Samuli Ripatti | Institute for Molecular Medicine Finland (FIMM), HiLIFE, University of Helsinki, Helsinki, Finland |
| Mark Daly | Institute for Molecular Medicine, Finland (FIMM), HiLIFE, University of Helsinki, Helsinki, Finland; Broad Institute of MIT and Harvard; Massachusetts General Hospital |
| Juha Karjalainen | Institute for Molecular Medicine Finland (FIMM), HiLIFE, University of Helsinki, Helsinki, Finland |
| Aki Havulinna | Institute for Molecular Medicine Finland (FIMM), HiLIFE, University of Helsinki, Helsinki, Finland; Finnish Institute for Health and Welfare (THL), Helsinki, Finland |
| Juha Mehtonen | Institute for Molecular Medicine Finland (FIMM), HiLIFE, University of Helsinki, Helsinki, Finland |
| Priit Palta | Institute for Molecular Medicine Finland (FIMM), HiLIFE, University of Helsinki, Helsinki, Finland |
| Shabbeer Hassan | Institute for Molecular Medicine Finland (FIMM), HiLIFE, University of Helsinki, Helsinki, Finland |
| Pietro Della Briotta Parolo | Institute for Molecular Medicine Finland (FIMM), HiLIFE, University of Helsinki, Helsinki, Finland |
| Wei Zhou | Broad Institute, Cambridge, MA, United States |
| Mutaamba Maasha | Broad Institute, Cambridge, MA, United States |
| Shabbeer Hassan | Institute for Molecular Medicine Finland (FIMM), HiLIFE, University of Helsinki, Helsinki, Finland |
| Susanna Lemmelä | Institute for Molecular Medicine Finland (FIMM), HiLIFE, University of Helsinki, Helsinki, Finland |
| Manuel Rivas | University of Stanford, Stanford, CA, United States |
| Aarno Palotie | Institute for Molecular Medicine Finland (FIMM), HiLIFE, University of Helsinki, Helsinki, Finland |
| Aoxing Liu | Institute for Molecular Medicine Finland (FIMM), HiLIFE, University of Helsinki, Helsinki, Finland |
| Arto Lehisto | Institute for Molecular Medicine Finland (FIMM), HiLIFE, University of Helsinki, Helsinki, Finland |
| Andrea Ganna | Institute for Molecular Medicine Finland (FIMM), HiLIFE, University of Helsinki, Helsinki, Finland |
| Vincent Llorens | Institute for Molecular Medicine Finland (FIMM), HiLIFE, University of Helsinki, Helsinki, Finland |
| Hannele Laivuori | Institute for Molecular Medicine Finland (FIMM), HiLIFE, University of Helsinki, Helsinki, Finland |
| Taru Tukiainen | Institute for Molecular Medicine Finland (FIMM), HiLIFE, University of Helsinki, Helsinki, Finland |
| Mary Pat Reeve | Institute for Molecular Medicine Finland (FIMM), HiLIFE, University of Helsinki, Helsinki, Finland |
| Henrike Heyne | Institute for Molecular Medicine Finland (FIMM), HiLIFE, University of Helsinki, Helsinki, Finland |
| Nina Mars | Institute for Molecular Medicine Finland (FIMM), HiLIFE, University of Helsinki, Helsinki, Finland |
| Joel Rämö | Institute for Molecular Medicine Finland (FIMM), HiLIFE, University of Helsinki, Helsinki, Finland |
| Elmo Saarentaus | Institute for Molecular Medicine Finland (FIMM), HiLIFE, University of Helsinki, Helsinki, Finland |
| Hanna Ollila | Institute for Molecular Medicine Finland (FIMM), HiLIFE, University of Helsinki, Helsinki, Finland |
| Rodos Rodosthenous | Institute for Molecular Medicine Finland (FIMM), HiLIFE, University of Helsinki, Helsinki, Finland |
| Satu Strausz | Institute for Molecular Medicine Finland (FIMM), HiLIFE, University of Helsinki, Helsinki, Finland |
| Tuula Palotie | University of Helsinki and Hospital District of Helsinki and Uusimaa, Helsinki, Finland |
| Kimmo Palin | University of Helsinki, Helsinki, Finland |
| Javier Garcia-Tabuenca | University of Tampere, Tampere, Finland |
| Harri Siirtola | University of Tampere, Tampere, Finland |
| Tuomo Kiiskinen | Institute for Molecular Medicine Finland (FIMM), HiLIFE, University of Helsinki, Helsinki, Finland |
| Jiwoo Lee | Institute for Molecular Medicine Finland (FIMM), HiLIFE, University of Helsinki, Helsinki, Finland; Broad Institute, Cambridge, MA, United States |
| Kristin Tsuo | Institute for Molecular Medicine Finland (FIMM), HiLIFE, University of Helsinki, Helsinki, Finland; Broad Institute, Cambridge, MA, United States |
| Amanda Elliott | Institute for Molecular Medicine Finland (FIMM), HiLIFE, University of Helsinki, Helsinki, Finland; Broad Institute, Cambridge, MA, USA and Massachusetts General Hospital, Boston, MA, USA |
| Kati Kristiansson | THL Biobank / Finnish Institute for Health and Welfare (THL), Helsinki, Finland |
| Mikko Arvas | Finnish Red Cross Blood Service / Finnish Hematology Registry and Clinical Biobank, Helsinki, Finland |
| Kati Hyvärinen | Finnish Red Cross Blood Service, Helsinki, Finland |
| Jarmo Ritari | Finnish Red Cross Blood Service, Helsinki, Finland |
| Olli Carpén | Helsinki Biobank / Helsinki University and Hospital District of Helsinki and Uusimaa, Helsinki |
| Johannes Kettunen | Northern Finland Biobank Borealis / University of Oulu / Northern Ostrobothnia Hospital District, Oulu, Finland |
| Katri Pylkäs | University of Oulu, Oulu, Finland |
| Eeva Sliz | University of Oulu, Oulu, Finland |
| Minna Karjalainen | University of Oulu, Oulu, Finland |
| Tuomo Mantere | Northern Finland Biobank Borealis / University of Oulu / Northern Ostrobothnia Hospital District, Oulu, Finland |
| Eeva Kangasniemi | Finnish Clinical Biobank Tampere / University of Tampere / Pirkanmaa Hospital District, Tampere, Finland |
| Sami Heikkinen | University of Eastern Finland, Kuopio, Finland |
| Arto Mannermaa | Biobank of Eastern Finland / University of Eastern Finland / Northern Savo Hospital District, Kuopio, Finland |
| Eija Laakkonen | University of Jyväskylä, Jyväskylä, Finland |
| Nina Pitkänen | Auria Biobank / University of Turku / Hospital District of Southwest Finland, Turku, Finland |
| Samuel Lessard | Translational Sciences, Sanofi R&D, Framingham, MA, USA |
| Clément Chatelain | Translational Sciences, Sanofi R&D, Framingham, MA, USA |
| Lila Kallio | Auria Biobank / University of Turku / Hospital District of Southwest Finland, Turku, Finland |
| Tiina Wahlfors | THL Biobank / Finnish Institute for Health and Welfare (THL), Helsinki, Finland |
| Jukka Partanen | Finnish Red Cross Blood Service / Finnish Hematology Registry and Clinical Biobank, Helsinki, Finland |
| Eero Punkka | Helsinki Biobank / Helsinki University and Hospital District of Helsinki and Uusimaa, Helsinki |
| Raisa Serpi | Northern Finland Biobank Borealis / University of Oulu / Northern Ostrobothnia Hospital District, Oulu, Finland |
| Sanna Siltanen | Finnish Clinical Biobank Tampere / University of Tampere / Pirkanmaa Hospital District, Tampere, Finland |
| Veli-Matti Kosma | Biobank of Eastern Finland / University of Eastern Finland / Northern Savo Hospital District, Kuopio, Finland |
| Teijo Kuopio | Central Finland Biobank / University of Jyväskylä / Central Finland Health Care District, Jyväskylä, Finland |
| Anu Jalanko | Institute for Molecular Medicine Finland (FIMM), HiLIFE, University of Helsinki, Helsinki, Finland |
| Huei-Yi Shen | Institute for Molecular Medicine Finland (FIMM), HiLIFE, University of Helsinki, Helsinki, Finland |
| Risto Kajanne | Institute for Molecular Medicine Finland (FIMM), HiLIFE, University of Helsinki, Helsinki, Finland |
| Mervi Aavikko | Institute for Molecular Medicine Finland (FIMM), HiLIFE, University of Helsinki, Helsinki, Finland |
| Helen Cooper | Institute for Molecular Medicine Finland (FIMM), HiLIFE, University of Helsinki, Helsinki, Finland |
| Denise Öller | Institute for Molecular Medicine Finland (FIMM), HiLIFE, University of Helsinki, Helsinki, Finland |
| Rasko Leinonen | Institute for Molecular Medicine Finland (FIMM), HiLIFE, University of Helsinki, Helsinki, Finland; European Molecular Biology Laboratory, European Bioinformatics Institute, Cambridge, UK |
| Henna Palin | Finnish Clinical Biobank Tampere / University of Tampere / Pirkanmaa Hospital District, Tampere, Finland |
| Malla-Maria Linna | Helsinki Biobank / Helsinki University and Hospital District of Helsinki and Uusimaa, Helsinki |
| Mitja Kurki | Institute for Molecular Medicine Finland (FIMM), HiLIFE, University of Helsinki, Helsinki, Finland; Broad Institute, Cambridge, MA, United States |
| Juha Karjalainen | Institute for Molecular Medicine Finland (FIMM), HiLIFE, University of Helsinki, Helsinki, Finland |
| Pietro Della Briotta Parolo | Institute for Molecular Medicine Finland (FIMM), HiLIFE, University of Helsinki, Helsinki, Finland |
| Arto Lehisto | Institute for Molecular Medicine Finland (FIMM), HiLIFE, University of Helsinki, Helsinki, Finland |
| Juha Mehtonen | Institute for Molecular Medicine Finland (FIMM), HiLIFE, University of Helsinki, Helsinki, Finland |
| Wei Zhou | Broad Institute, Cambridge, MA, United States |
| Masahiro Kanai | Broad Institute, Cambridge, MA, United States |
| Mutaamba Maasha | Broad Institute, Cambridge, MA, United States |
| Zhili Zheng | Broad Institute, Cambridge, MA, United States |
| Hannele Laivuori | Institute for Molecular Medicine Finland (FIMM), HiLIFE, University of Helsinki, Helsinki, Finland |
| Aki Havulinna | Institute for Molecular Medicine Finland (FIMM), HiLIFE, University of Helsinki, Helsinki, Finland; Finnish Institute for Health and Welfare (THL), Helsinki, Finland |
| Susanna Lemmelä | Institute for Molecular Medicine Finland (FIMM), HiLIFE, University of Helsinki, Helsinki, Finland |
| Tuomo Kiiskinen | Institute for Molecular Medicine Finland (FIMM), HiLIFE, University of Helsinki, Helsinki, Finland |
| L. Elisa Lahtela | Institute for Molecular Medicine Finland (FIMM), HiLIFE, University of Helsinki, Helsinki, Finland |
| Mari Kaunisto | Institute for Molecular Medicine Finland (FIMM), HiLIFE, University of Helsinki, Helsinki, Finland |
| Elina Kilpeläinen | Institute for Molecular Medicine Finland (FIMM), HiLIFE, University of Helsinki, Helsinki, Finland |
| Timo P. Sipilä | Institute for Molecular Medicine Finland (FIMM), HiLIFE, University of Helsinki, Helsinki, Finland |
| Oluwaseun Alexander Dada | Institute for Molecular Medicine Finland (FIMM), HiLIFE, University of Helsinki, Helsinki, Finland |
| Awaisa Ghazal | Institute for Molecular Medicine Finland (FIMM), HiLIFE, University of Helsinki, Helsinki, Finland |
| Anastasia Kytölä | Institute for Molecular Medicine Finland (FIMM), HiLIFE, University of Helsinki, Helsinki, Finland |
| Rigbe Weldatsadik | Institute for Molecular Medicine Finland (FIMM), HiLIFE, University of Helsinki, Helsinki, Finland |
| Sanni Ruotsalainen | Institute for Molecular Medicine Finland (FIMM), HiLIFE, University of Helsinki, Helsinki, Finland |
| Kati Donner | Institute for Molecular Medicine Finland (FIMM), HiLIFE, University of Helsinki, Helsinki, Finland |
| Timo P. Sipilä | Institute for Molecular Medicine Finland (FIMM), HiLIFE, University of Helsinki, Helsinki, Finland |
| Anu Loukola | Helsinki Biobank / Helsinki University and Hospital District of Helsinki and Uusimaa, Helsinki |
| Päivi Laiho | THL Biobank / Finnish Institute for Health and Welfare (THL), Helsinki, Finland |
| Tuuli Sistonen | THL Biobank / Finnish Institute for Health and Welfare (THL), Helsinki, Finland |
| Essi Kaiharju | THL Biobank / Finnish Institute for Health and Welfare (THL), Helsinki, Finland |
| Markku Laukkanen | THL Biobank / Finnish Institute for Health and Welfare (THL), Helsinki, Finland |
| Elina Järvensivu | THL Biobank / Finnish Institute for Health and Welfare (THL), Helsinki, Finland |
| Sini Lähteenmäki | THL Biobank / Finnish Institute for Health and Welfare (THL), Helsinki, Finland |
| Lotta Männikkö | THL Biobank / Finnish Institute for Health and Welfare (THL), Helsinki, Finland |
| Regis Wong | THL Biobank / Finnish Institute for Health and Welfare (THL), Helsinki, Finland |
| Auli Toivola | THL Biobank / Finnish Institute for Health and Welfare (THL), Helsinki, Finland |
| Minna Brunfeldt | THL Biobank / Finnish Institute for Health and Welfare (THL), Helsinki, Finland |
| Hannele Mattsson | THL Biobank / Finnish Institute for Health and Welfare (THL), Helsinki, Finland |
| Kati Kristiansson | THL Biobank / Finnish Institute for Health and Welfare (THL), Helsinki, Finland |
| Susanna Lemmelä | Institute for Molecular Medicine Finland (FIMM), HiLIFE, University of Helsinki, Helsinki, Finland |
| Sami Koskelainen | THL Biobank / Finnish Institute for Health and Welfare (THL), Helsinki, Finland |
| Tero Hiekkalinna | THL Biobank / Finnish Institute for Health and Welfare (THL), Helsinki, Finland |
| Teemu Paajanen | THL Biobank / Finnish Institute for Health and Welfare (THL), Helsinki, Finland |
| Priit Palta | Institute for Molecular Medicine Finland (FIMM), HiLIFE, University of Helsinki, Helsinki, Finland |
| Shuang Luo | Institute for Molecular Medicine Finland (FIMM), HiLIFE, University of Helsinki, Helsinki, Finland |
| Tarja Laitinen | Pirkanmaa Hospital District, Tampere, Finland |
| Mary Pat Reeve | Institute for Molecular Medicine Finland (FIMM), HiLIFE, University of Helsinki, Helsinki, Finland |
| Shanmukha Sampath Padmanabhuni | Institute for Molecular Medicine Finland (FIMM), HiLIFE, University of Helsinki, Helsinki, Finland |
| Marianna Niemi | University of Tampere, Tampere, Finland |
| Harri Siirtola | University of Tampere, Tampere, Finland |
| Javier Gracia-Tabuenca | University of Tampere, Tampere, Finland |
| Mika Helminen | University of Tampere, Tampere, Finland |
| Tiina Luukkaala | University of Tampere, Tampere, Finland |
| Iida Vähätalo | University of Tampere, Tampere, Finland |
| Jyrki Tammerluoto | Institute for Molecular Medicine Finland (FIMM), HiLIFE, University of Helsinki, Helsinki, Finland |
| Marco Hautalahti | Finnish Biobank Cooperative - FINBB |
| Johanna Mäkelä | Finnish Biobank Cooperative - FINBB |
| Sarah Smith | Finnish Biobank Cooperative - FINBB |
| Tom Southerington | Finnish Biobank Cooperative - FINBB |
| Petri Lehto | Finnish Biobank Cooperative - FINBB |

# Supplementary tables

**Supplementary table 1. Correlations of 4αHC and blood pressure**

|  | SBP early pregnancy | DBP early pregnancy | Highest SBP before 20 weeks of gestation | Highest DBP before 20 weeks of gestation | SBP at the time of sampling | DBP at the time of sampling | BP MAP 1st visit |
| --- | --- | --- | --- | --- | --- | --- | --- |
| Normal pregnancies 4αHC | 0.123 (p=0.46) n=38 | -0.141 (p=0.40) n=38 | -0.047 (p=0.78) n=38 | -0.210 (p=0.21) n=38 | -0.132 (p=0.43) n=38 | -0.084 (p=0.62) n=38 | -0.098 (p=0.56) n=38 |
| Pre-eclampsia 4αHC | 0.070 (p=0.41) n=143 | 0.075 (p=0.37) n=143 | 0.059 (p=0.49) n=142 | 0.096 (p=0.26) n=142 | -0.017 (p=0.84) n=144 | 0.059 (p=0.48) n=144 | 0.076 (p=0.37) n=143 |
| All pregnancies 4αHC | 0.101 (p=0.17) n=181 | 0.074 (p=0.32) n=181 | 0.049 (p=0.52) n=180 | 0.042 (p=0.58) n=180 | 0.060 (p=0.42) n=182 | 0.089 (p=0.23) n=182 | 0.089 (p=0.24) n=181 |

Spearman’s rank correlation was employed as the statistical method. 4αHC 4α-hydroxycholesterol; SBP systolic blood pressure; DBP diastolic blood pressure; BMI body mass index; MAP mean arterial pressure. We considered p <0.05 statistically significant.

**Supplementary table 2. Correlations of 4αHC and maternal and perinatal characteristics**

|  | Maternal BMI at the start of pregnancy | Maternal BMI at term | Maternal weight gain | Birth weight of the child | Weight of the placenta | Gestational age at the time of sampling | Maternal age at delivery |
| --- | --- | --- | --- | --- | --- | --- | --- |
| Normal pregnancies 4αHC | -0.021 (p=0.90) n=38 | 0.007 (p=0.97) n=38 | 0.134 (p=0.42) n=38 | 0.214 (p=0.20) n=38 | 0.128 (p=0.45) n=37 | 0.107 (p=0.64) n=22 | 0.088 (p=0.60) n=38 |
| Pre-eclampsia 4αHC | -0.096 (p=0.25) n=144 | -0.110 (p=0.19) n=144 | 0.011 (p=0.90) n=144 | -0.009 (p=0.92) n=144 | 0.024 (p=0.78) n=138 | -0.160 (p=0.20) n=66 | 0.134 (p=0.11) n=144 |
| All pregnancies 4αHC | -0.079 (p=0.29) n=182 | -0.085 (p=0.26) n=182 | 0.045 (p=0.54) n=182 | -0.023 (p=0.76) n=182 | 0.025 (p=0.75) n=175 | -0.280 **(p=0.008)** n=88 | 0.128 (p=0.086) n=182 |

Spearman’s rank correlation was employed as the statistical method. 4αHC 4α-hydroxycholesterol; SBP systolic blood pressure; DBP diastolic blood pressure; BMI body mass index; MAP mean arterial pressure. We considered p <0.05 statistically significant.

**Supplementary table 3. LXR target genes (alias)**

| LXRα (NR1H3) | APOA4 |
| --- | --- |
| LXRβ (NR1H2) | APOC1 |
| SREBF1 | APOC2 |
| PTX3 | APOC4 |
| CCR7 | APOD |
| RXRA | ARG2 |
| RXRB | EEPD1 |
| RXRC | FABP6 |
| ACACA | IL18 |
| ABCA1 | NPC1 |
| ABCG1 | NPC1L1 |
| ABCG5 | NPC2 |
| ABCG8 | NR1D1 |
| CETP | PLTP |
| IDOL (MYLIP) | RARA |
| APOE | SCARB1 |
| LPL | SULT2A1 |
| ACSL3 | TLR4 |
| CD36 | TNF |
| LPCAT3 | UGT1A3 |
| LDLR | VEGFA |
| MLXIPL | LBP |
| MLX | MAFB |
| CYP7A1 | TGM2 |
| ADIPOR1 | MMP9 |
| ADIPOR2 | ACACB |
| ANGPTL3 | FAS |

# Supplementary Figure legends

Supplementary Figure 1. Associations of variants in the NR1H2 (LXRβ) locus with (A) pregnancy hypertension, (B) with pre-eclampsia, (C) with pre-eclampsia or eclampsia and (D) with pre-existing hypertension.

Supplementary Figure 2. Associations of variants in the NR1H3 (LXRα) locus (A) with pregnancy hypertension, (B) with pre-eclampsia, (C) with pre-eclampsia or eclampsia and (D) with pre-existing hypertension.

Supplementary Figure 3. Gene expression of LXR pathway genes between PE and NP samples. Shown are relative expression levels of 22 LXR pathway genes in PE (red) and NP (black) samples. Columns represent samples and rows genes. The colour scale indicates normalized expression, with red showing relative overexpression and blue indicating relative underexpression.

Supplementary Figure 4. Significant correlations of LXR pathway genes with plasma oxysterols (4αHC and 4βHC). Normal pregnancies (NP) are presented with black dots and PE pregnancies are presented with red dots. Shown are significant correlations between LXR target genes and plasma 4αHC and 4βHC levels (padj < 0.05). (A) LPL with 4αHC, (B) LPL with 4βHC, (C) APOC1 with 4αHC, (D) APOC1 with 4βHC. (E) APOD with 4αHC, (F) APOD with 4βHC, (G) CD36 with 4αHC and (H) CD36 with 4βHC
