# Supplementary figures and images for: Role of oxysterol 4β-hydroxycholesterol and liver X receptor alleles in pre-eclampsia
Source: Ann Med. 2025 Apr 29;57(1):2495763. doi: 10.1080/07853890.2025.2495763 (PMC12042236; doi:10.1080/07853890.2025.2495763)

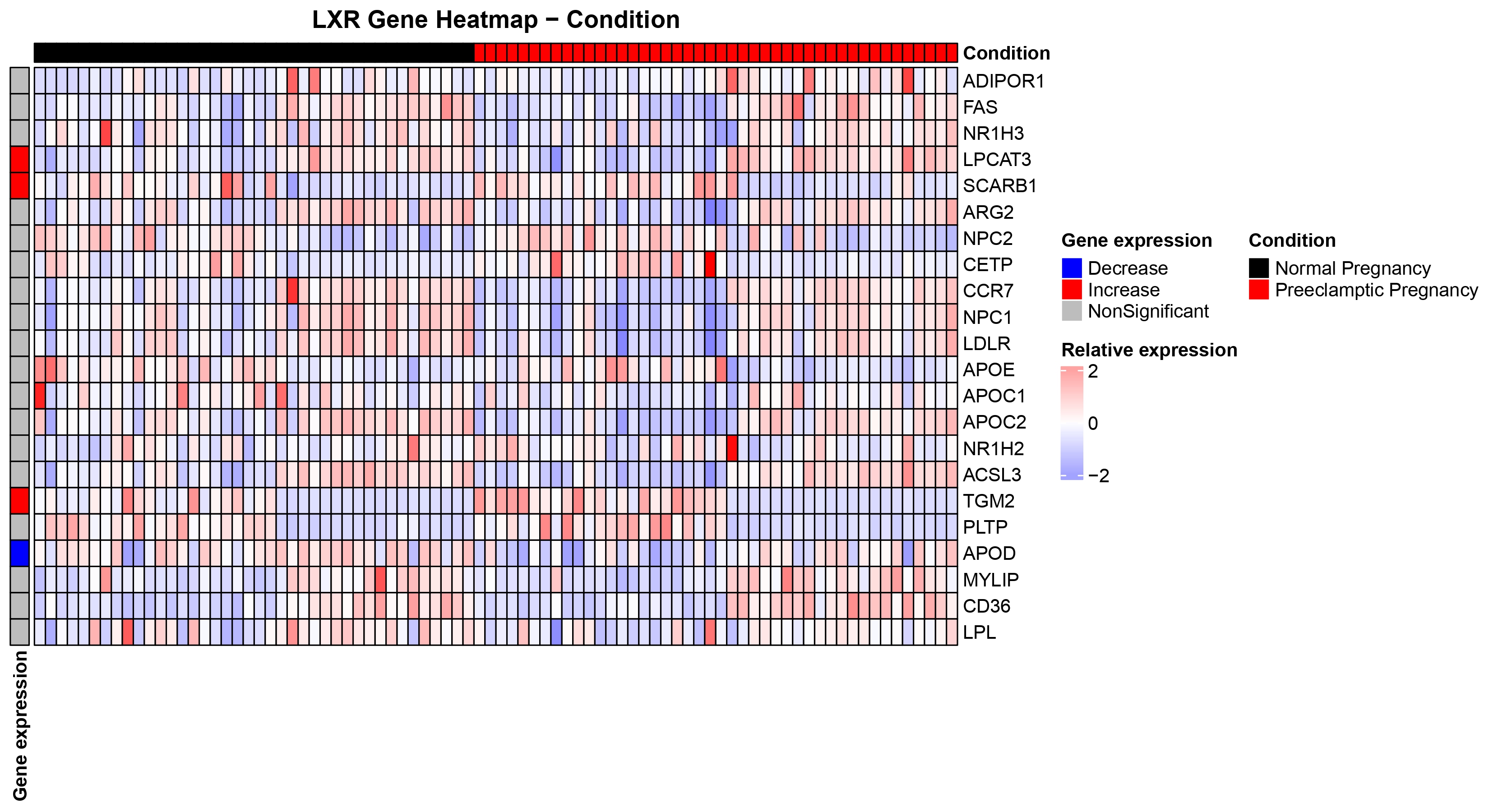

Supplement: Supplemental Material [file IANN_A_2495763_SM9417.zip › Suppl/Supplementary Figure 3.jpg]

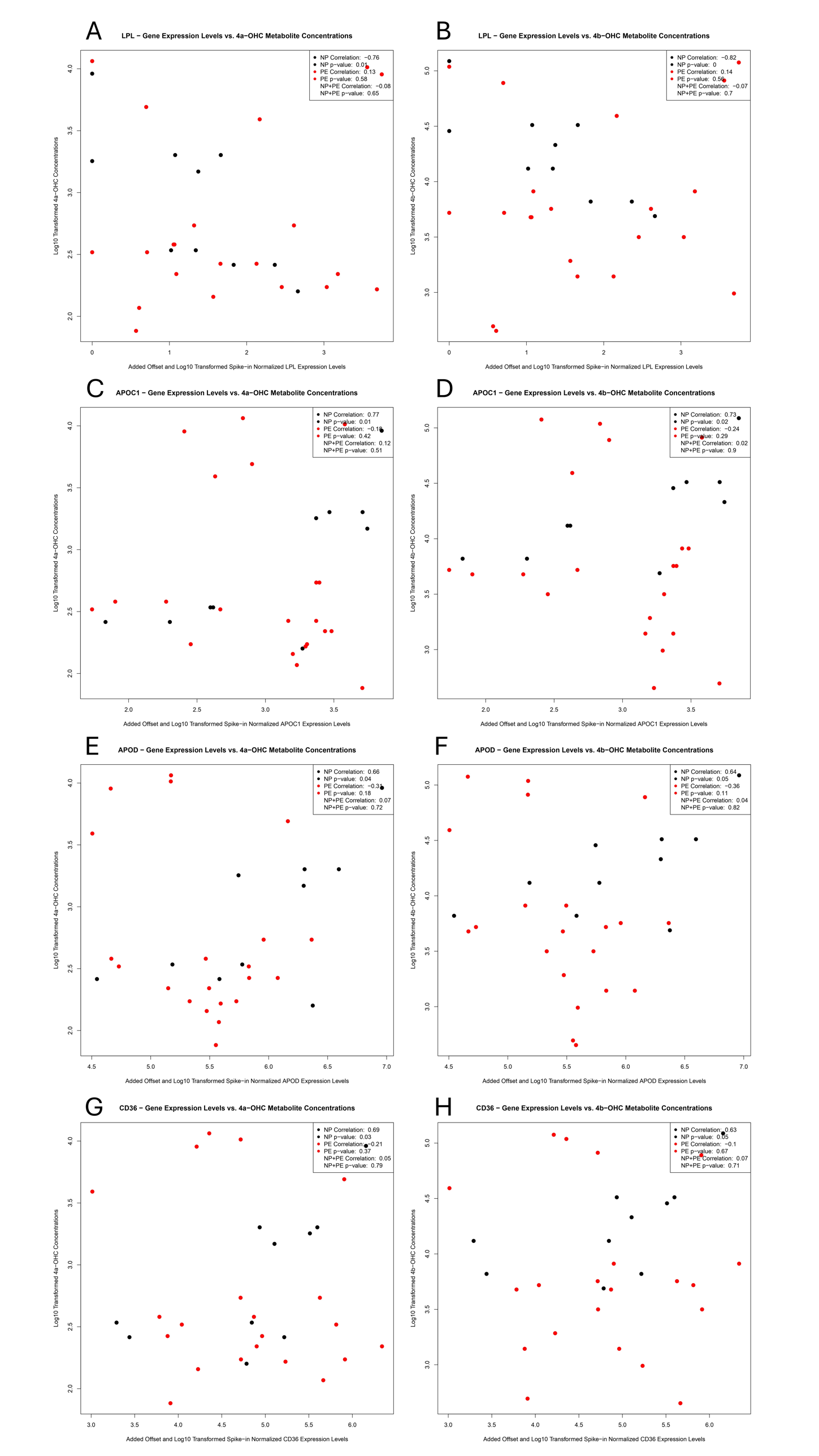

Supplement: Supplemental Material [file IANN_A_2495763_SM9417.zip › Suppl/Supplementary Figure 4.jpg]
